# Ligand-bound glutamine binding protein assumes multiple metastable binding sites with different binding affinities

**DOI:** 10.1038/s42003-020-01149-z

**Published:** 2020-08-03

**Authors:** Lu Zhang, Shaowen Wu, Yitao Feng, Dan Wang, Xilin Jia, Zhijun Liu, Jianwei Liu, Wenning Wang

**Affiliations:** 1 State Key Laboratory of Structural Chemistry, Fujian Institute of Research on the Structure of Matter, Chinese Academy of Sciences, Fuzhou, Fujian China; 2 Department of Chemistry, Institutes of Biomedical Sciences, Multiscale Research Institute of Complex Systems, Fudan University, Shanghai, China; 3 University of Chinese Academy of Sciences, Beijing, China; 4 National Center for Protein Science, Shanghai Institute of Biochemistry and Cell Biology, Chinese Academy of Sciences, Shanghai, China

**Keywords:** Computational biophysics, Single-molecule biophysics

## Abstract

Protein dynamics plays key roles in ligand binding. However, the microscopic description of conformational dynamics-coupled ligand binding remains a challenge. In this study, we integrate molecular dynamics simulations, Markov state model (MSM) analysis and experimental methods to characterize the conformational dynamics of ligand-bound glutamine binding protein (GlnBP). We show that ligand-bound GlnBP has high conformational flexibility and additional metastable binding sites, presenting a more complex energy landscape than the scenario in the absence of ligand. The diverse conformations of GlnBP demonstrate different binding affinities and entail complex transition kinetics, implicating a concerted ligand binding mechanism. Single molecule fluorescence resonance energy transfer measurements and mutagenesis experiments are performed to validate our MSM-derived structure ensemble as well as the binding mechanism. Collectively, our study provides deeper insights into the protein dynamics-coupled ligand binding, revealing an intricate regulatory network underlying the apparent binding affinity.

## Abstract (web summary)

Zhang, Wu, Feng et al. show that ligand-bound glutamine binding protein assumes multiple metastable binding sites, presenting a more dynamic energy landscape than its ligand-free form. This study provides insights into the ligand-binding mechanisms coupled with protein dynamics that underly the apparent binding affinity.

## Introduction

Elucidation of protein dynamics and understanding how it is coupled with molecular recognition offer great insight into molecular control of cellular function^1–7^. Although the modulation role of protein dynamics in ligand binding has seen much attention, a full microscopic description of the coupling between conformational dynamics and ligand binding is still challenging. Fundamentally, protein structural plasticity entails plenty of metastable states of protein in both ligand unbound and ligand-bound forms, which may not be easily accessible by experimental techniques. The diversity of structural features, energetics and kinetics of these states could result in binding thermodynamics and kinetics far more complicated than the simple models such as induced-fit and conformational selection^8–16^. Therefore, dissecting the detailed conformational dynamics and energetics underlying ligand recognition of proteins is critical for understanding the modulation of affinity and related drug design.

Periplasmic binding proteins (PBPs) provide a typical model system to investigate the impact of protein dynamics on the ligand binding^17^. They are a superfamily of proteins responsible for binding and delivering substrates across the cell membrane by collaborating with the cognate ATP-binding cassette transporters^17,18^. X-ray crystallographic studies, nuclear magnetic resonance (NMR), single molecule fluorescence resonance energy transfer (smFRET) and phosphorescence spectroscopy have been applied to investigate the conformational dynamics of PBPs^13,19–31^. Glutamine binding protein (GlnBP) is a PBP that assists L-glutamine uptake in *Escherichia coli* and it has a similar three-dimensional structural scaffold to other PBPs^18–20,32–37^. Crystal structures^19,20^ and NMR paramagnetic relaxation enhancement studies^25,28,30^ have suggested that ligand-bound GlnBP only adopts closed conformation while ligand-free GlnBP is solely in the open conformation, supporting the conventional Venus Flytrap model. However, phosphorescence spectroscopy, NMR residual dipolar couplings, and smFRET experiments suggested inter-domain dynamics in ligand-free GlnBP^24,30,31^, raising the possibility that conformational selection could play a role in its molecular recognition. Complementary to experimental techniques, molecular dynamics (MD) simulations and Markov state models (MSMs) method have become popular tools to decipher the dynamic structural ensemble of proteins at both atomic resolution and biologically relevant timescales^38–53^. They have been adopted to discover metastable state and provide the kinetic information for molecular recognition for PBPs^31,54–58^ and elucidated multiple metastable states for ligand-free GlnBP^31^. Moreover, computational coarse-grained model proposed GlnBP could undergo large-scale closed-to-open transition^59^. These studies demonstrate the conformational complexity of GlnBP and pave the way for elucidating its ligand binding mechanism. However, there are still critical questions remain largely unsolved and exploring the conformational dynamics of ligand-bound-GlnBP is prerequisite for fully understanding its molecular recognition mechanism.

In this work, we have combined MSM based on extensive MD simulations, smFRET, and site-directed mutagenesis to obtain a comprehensive insight into the conformational dynamics of ligand-bound GlnBP and elucidate its role in molecular recognition. Both our experimental data and computational models suggest that ligand-bound GlnBP has complex conformational dynamics in solution. Besides the inter-domain motion implicated by the crystal structures, we have also observed ligand migration between two domains and found a binding pocket in the small domain. The inter-state transitions estimated by the kinetic network model, as well as the different binding affinities of multiple conformational states together suggest an intricate interplay between the conformational dynamics and ligand binding, which were further validated by site-directed mutagenesis and smFRET experiments. Taken together, our work offers not only a deeper insight into the complex conformational diversity of ligand-bound GlnBP, but also provides direct evidence that protein dynamics is essential for ligand binding.

## Results

### Ligand-bound GlnBP shows multiple ligand binding sites

**Fig. 1:**
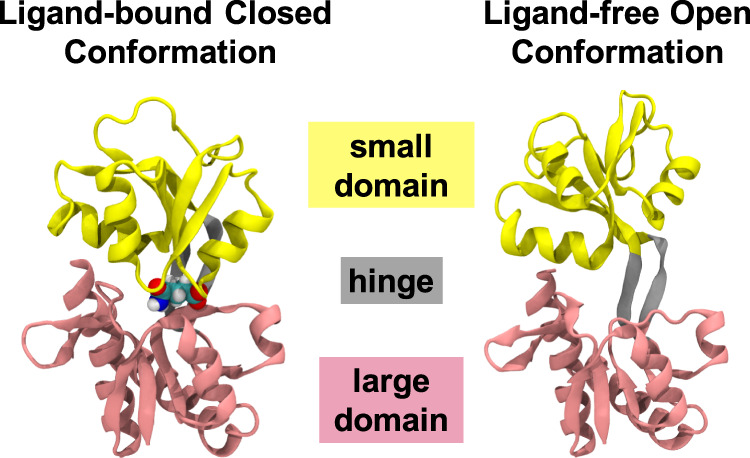
Crystal structure of the ligand-bound closed and ligand-free open GlnBP. The small domain, the large domain and the hinge is shown in yellow, pink and grey, respectively. In the ligand-bound closed conformation, the ligand is shown in spheres.

**Fig. 2:**
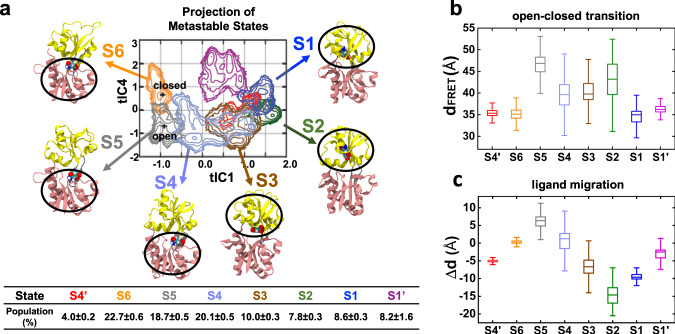
MSM analysis elucidates eight macrostates, demonstrating not only the open-closed transitions, but also the ligand displacement between two domains. **a** Projection of macrostates onto two tICs. Representative conformations of the six states on the major transition pathway are shown with the ligand-binding region highlighted in black circle. MSM-derived populations of macrostates are labeled. The crosses label the projection of the crystal structures (Fig. 1). **b** The open-closed transition of protein was measured as distance between Cα atoms of Thr59 and Thr130. **c** The ligand migration was estimated by its relative distance to the large/small domain. The averaged pairwise distances between the heavy atoms of the ligand and the Cα atoms in each domain were calculated. Δd equals to the d(ligand-small domain)—d(ligand-large domain). In **b**, **c**, the box plot for each macrostate was calculated based on all the MD conformations belonging to the specific state.

Crystal structures have demonstrated that GlnBP is composed of two globular domains, the large domain (protein residues 5–84 and 186–224) and the small domain (protein residues 90–180), connected by a hinge region (protein residues 85–89 and 181 and 185), and the substrate glutamine binds at the domain surface (Fig. 1)^19,20^. However, our MD simulations of ligand-bound GlnBP have sampled a broad conformational space beyond the crystal structures. The simulation was initiated from the crystal structure^20^ of ligand-bound GlnBP (closed conformation), followed by several rounds of simulations (~60 µs) to reach a converged conformational space (Supplementary Fig. 1a, see “Methods” for details). To gain insight into the underlying thermodynamics and kinetics, we constructed the MSM^38–46^ based on the MD trajectories using a recently developed algorithm time-structure-based independent component analysis (tICA)^44,45^ (see “Methods” for details). In particular, the MD conformational space was decomposed into ~700 microstates according to the kinetic similarity. To examine the underlying molecular mechanism, the microstates were further grouped into eight macrostates, which represent local minimums in the free energy landscape and their inter-state transitions are separated by energy barriers. Besides the two states similar to the crystal structures^19,20^, another six macrostates were elucidated, which are well separated by not only the open-closed transitions, but also the ligand displacement between two domains (Fig. 2). The extent of opening was measured by the Cα–Cα distance between Thr59 (large domain) and Thr130 (small domain) (Fig. 2b). These two residues were chosen as they were used to attach fluorophore labels in the smFRET measurements (see below for details). Four of the eight states (S1, S6, S1′, and S4′) exhibit short average distances between 35 and 36 Å, and could be assigned as closed conformations. The S2 and S5 states could be assigned to open conformations due to the long average distance (43.1 Å for S2 and 46.1 Å for S5). The inter-residue distances of states S3 and S4 are moderate and thus were assigned as the “semi-closed” states. Examination of the representative structures of the eight macrostates revealed that the ligand could bind at different sites on GlnBP. In state S4, S5, and S6, the binding site is at the large domain. In states S1, S2, and S3, the ligand binds at the small domain (Fig. 2a), while the ligand binds at the interface between the large and small domain in states S1′ and S4′. This assignment of binding site was further confirmed by the relative distance between the ligand and two domains (Fig. 2c). Interestingly, we notice that although the tICs generated by tICA^44,45^ are usually a mixture of various conformational changes, the ligand migration and protein opening-closing motion were well captured by the first and the fourth tICs (Fig. 2a), suggesting that these conformational changes indeed make obvious contributions to the ligand-bound GlnBP dynamics.

### Ligand-binding affinity correlates with protein dynamics

**Fig. 3:**
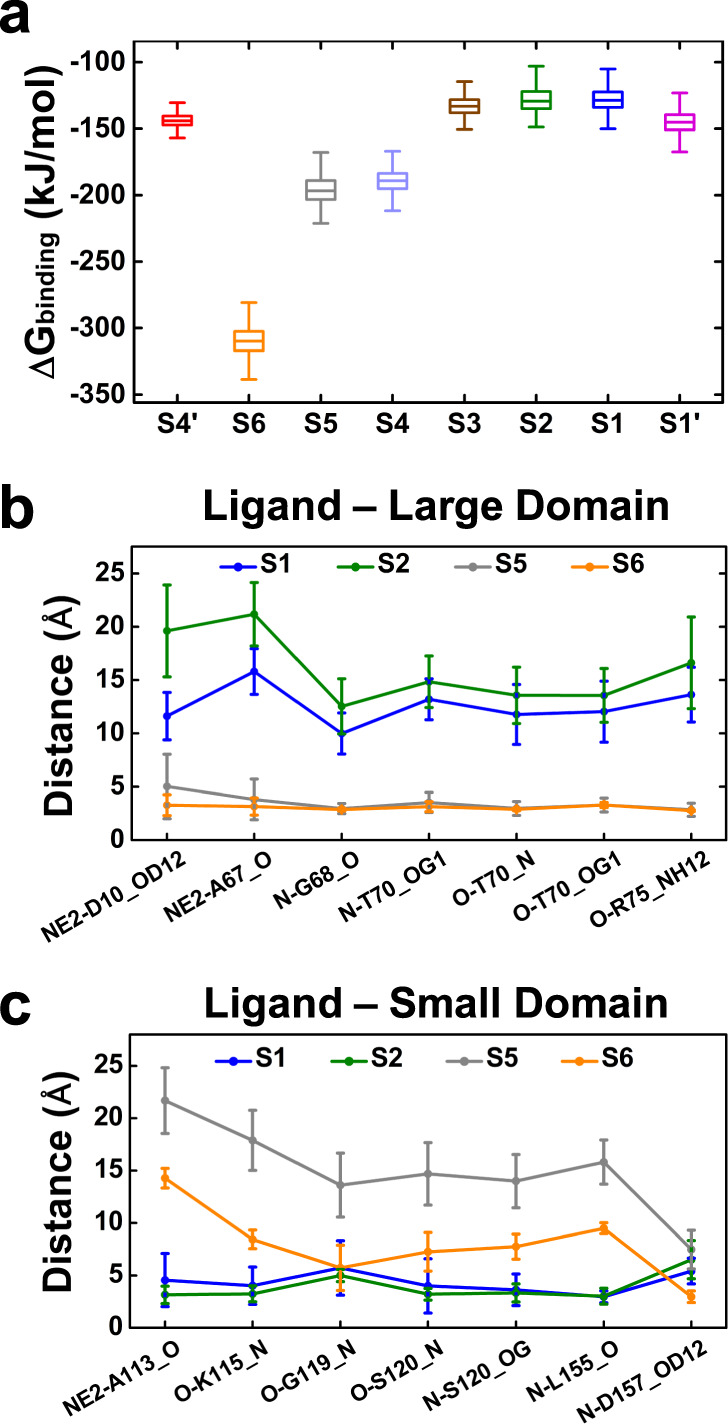
Ligand-binding affinity is correlated with the ligand-protein interactions. **a** Ligand-binding free energies of macrostates are estimated by MM-PBSA method. We performed 1000 interactions of calculations to achieve the statistics about the reported data. In each interaction, one random conformation was selected from each microstate and the binding free energy of each macrostate was estimated by the population weighted sum of the binding free energies of all the microstates belonging to the specific macrostate. Box plot represent the data distribution resulted from the 1000 times of calculations. **b** Interactions between ligand and the protein residues in the large domain. **c** Interactions between ligand and the small-domain residues. In **b**–**c**, the atoms used for distance calculations are labeled next to the x-axis and the minimum distance is reported when there are chemically equivalent atoms. The means and standard deviations of distances for each macrostate were calculated using all the MD conformations belonging to the specific state.

The ligand binding affinities for different macrostates were estimated by Poisson–Boltzmann surface area (MM-PBSA) method^60,61^ (see “Methods” for details). This method has been widely applied in areas of biomolecular studies and computational drug design to estimate the relative binding free energies^62,63^. We note the binding free energy of individual macrostate could not be measured by experimental method, but it can shed light on the relative binding strength between protein and ligand in each state. Accordingly, we found the ligand at large domain has higher binding affinity than it is at small domain (Fig. 3a). This matches with the observation that large-domain binding states S4, S5, and S6 have higher populations than the small-domain binding states S1, S2, and S3 (Fig. 2a). The calculated ligand binding affinities are also in line with the analysis of the ligand-protein interactions (Fig. 3). Ligand in S5 and S6 states show similar interactions with the large domain: the ligand’s alpha group forms stable interactions with the large-domain residues Gly68, Thr70, and Arg75 (averaged distance ~3.0 Å); the ligand’s side chain form a bit stronger interactions with Asp10 and Ala67 in S6 state than in S5 state (Fig. 3b). However, S6 has remarkably higher binding affinity than S5 (Fig. 3a), due to the binding site of S6 state is on the large-domain surface and additional interactions of the ligand with the small-domain residues Asp157 and Gly119 can be formed when protein adopts closed conformation (Fig. 3c). On a sharp contrary, S1 and S2 demonstrate different protein conformations (Fig. 2b), but they have similar ligand binding affinity (Fig. 3a). This can be explained by the observation that their binding pockets in the small domain are relatively buried (Supplementary Fig. 2) and ligand only interacts with protein residues in the small domain in these two states. Specifically, ligand in S1 and S2 states shows similar interactions with small-domain residues Ala113, Lys115, Gly119, Leu155, and Ser120 (Fig. 3c), while does not interact with the large-domain residues (Fig. 3b). Thus, the opening-closing motion would not perturb the ligand-protein interactions in S1 and S2 states. These observations altogether explain why S1 and S2 have similar ligand binding affinity (Fig. 3a), even though they demonstrate different protein conformations (Fig. 2b).

Furthermore, we found different binding sites can give rise to diverse binding affinities. As shown in Fig. 2b, S2 and S5 states show d_FRET_ of 43.1 ± 3.9 Å and 46.1 ± 3.2 Å, respectively, suggesting that their opening extent are similar. However, the binding affinities of S2 and S5 are notably different, with the binding affinity of S5 state obviously higher than that of S2 state (Fig. 3a). How these states are kinetically connected is a question worthy of exploring and this investigation would assist the understanding of the interplay between the protein dynamics and ligand binding.

### Protein dynamics modulates the kinetic transition network

**Fig. 4:**
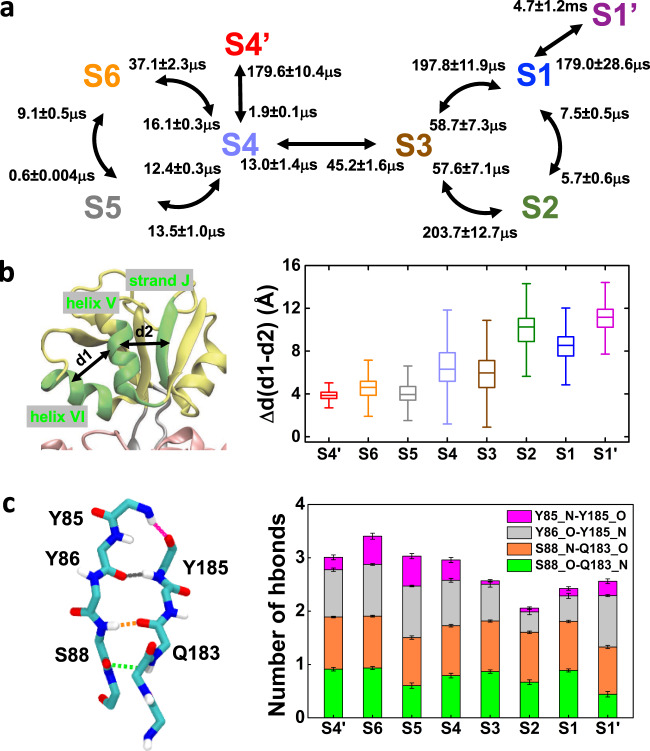
Modulation of the kinetic transition network by essential protein conformational changes. **a** Transition pathways elucidated by MSM, with mean first passage times alongside the arrows. **b** Structural rearrangement in the small domain manifested in Δd. d1 denotes the distance between helix VI (residues 160–168) and helix V (residues 138–146), while d2 for the helix V-strand J (residues 111–115) distance. Box plot for each macrostate represents the distribution of Δd. All the MD conformations belonging to the specific state were included in the statistics. **c** The rigidity of the hinge (residues 85–89 and 181 to 185) is represented by four pairs of hydrogen bonds. Each pair is shown with an individual color in the stacked bar. Bootstrapping algorithm with replacement was applied to estimate the statistical errors of the number of hydrogen bonds, in which 100 samples were generated and each sample contains 100 random conformations from the macrostate under investigation.

The kinetic transition network was elucidated by MSM (Fig. 4a), according to which the macrostates were roughly grouped into two clusters: one involves S1, S2, and S3 with ligand at the small domain, and the other involves S4, S5, and S6 with ligand at the large domain. The two clusters are connected by S3 and S4. S1′ and S4′ are two off-pathway states that only connect to S1 and S4, respectively. We found that the opening-closing transitions within cluster (S1↔S2 or S5↔S6) occur very fast (<10 μs), and there is no intermediate state during the transitions. Therefore, GlnBP could undergo quick opening-closing movement with the ligand bound at either the large or small domain. On the other hand, transitions through the semi-closed states S3 and S4 are slower, especially the S3→S1 and S3→S2 transitions. This suggests that besides the opening-closing motions, other conformational changes also determine the kinetics.

First, we found that the kinetics is partially determined by the structural rearrangements involving helix V (residues 138–146), helix VI (residues 160–168) and strand J (residues 111–115) in the small domain (Fig. 4b). The extent could be measured by the value of Δd = d1−d2 (d1 for the space between helix VI and helix V; d2 for that between helix V and strand J). As shown in Fig. 4b, in the semi-closed S3 and S4 states, the values of Δd are moderate. However, in S5, S6, and S4′ states, Δd values are small, implying the binding pocket of the small domain is almost locked. On the contrary, S1, S2, and S1′ states have obviously larger Δd values, thereby that pocket is wide enough to fully accommodate the ligand. This structural rearrangement plays an important role in not only modulating the kinetics but also to make enough space for ligand to diffuse into the binding site on the small domain.

Further structural analysis reveals the flexibility of the hinge region also tunes the kinetics. The rigidity of the hinge region is mainly contributed by four pairs of hydrogen bonds (Fig. 4c). As ligand migrates from large domain to small domain, the hinge becomes more flexible, mainly manifested by the decreasing bonding strength between the backbone of Y185 and that of Y85/Y86. We also notice although the off-pathway state S1′ has similar hydrogen bond number with S1 state, their hydrogen bonding patterns are different, involving weakening the hydrogen bond (S88_O-Q183_N) and simultaneously strengthening the hydrogen bond stability between Y86_O and Y185_N. This also rationalizes the slow off-pathway transition between S1 and S1′ states. The other off-pathway transition S4→S4′ is also relatively slow and this is due to the variation of Δd (Fig. 4b) as well as the expansion of a loop between strand J and helix IV to allocate the ligand (Supplementary Fig. 3).

Overall, by combining the kinetics derived from MSM analysis as well as detailed structural analysis, we have pinpointed the key structural features that determine the transition kinetics, providing the basis to understand the molecular recognition and manipulate the binding affinity of ligand-bound GlnBP.

### A concerted model for molecular recognition

To understand the ligand binding mechanism, a comparison between ligand-free and ligand-bound GlnBP was made. The conformational space of ligand-bound GlnBP explored herein shows that ligand binding expands the conformational space with respect to that of ligand-free GlnBP^31^ (Supplementary Fig. 4). First, we performed principal component analysis based on all Cα atoms using all the MD conformations of ligand-bound GlnBP and the same eigenvectors were adopted for projecting the ligand-free GlnBP conformations^31^. The projection of each macrostate sampled in ligand-bound GlnBP was compared to the overall projection of ligand-free GlnBP. Projection of S5 and S6 onto the top two components of principal component analysis shows considerable overlap with that of ligand-free GlnBP; while other states are partially (S2, S3, and S4) or not sampled (S1, S1′, and S4′) by ligand-free GlnBP (Supplementary Fig. 4a). Second, as the ligand binding induces the conformational rearrangement in the small domain (Fig. 4b), we also projected macrostates of ligand-bound GlnBP as well as ligand-free MD conformations onto the dimension of Δd. The comparison shows that states S3, S4, S5, and S6 remarkably overlap with ligand-free GlnBP, while the other states (S1, S1′, S2, and S4′) populate distinct regions (Supplementary Fig. 4b). Taken these results together, we may conclude that four macrostates (S3, S4, S5, and S6) of ligand-bound GlnBP have their counterparts in ligand-free GlnBP. The other four macrostates (S1, S1′, S2, and S4′) identified in ligand-bound GlnBP, which involve structural rearrangements of the small domain and/or remarkable inter-domain twist, are absent or very rarely populated in ligand-free GlnBP.

**Fig. 5:**
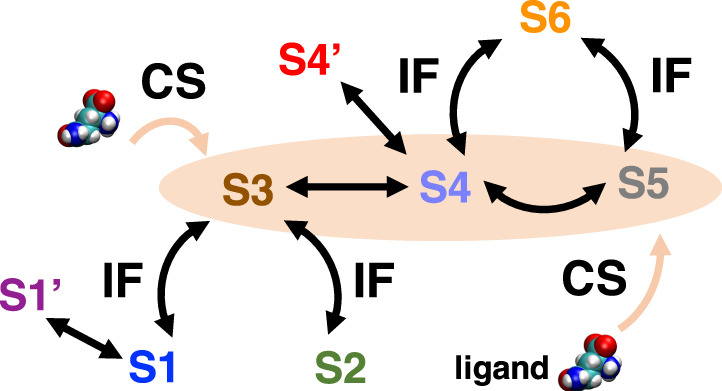
Ligand-binding mechanism for GlnBP. “Conformational selection” (CS) works for the initial selective binding to S5, S4 or S3 state, followed by “induced fit” (IF) for subsequent transitions to the states that barely exist in the absence of ligand.

Based on the structural features, the kinetic network and the comparison between ligand-bound and ligand-free GlnBP discussed above, we proposed a concerted mechanism of ligand binding (Fig. 5). Although the S6 state has the highest binding affinity and its conformation exists in the ligand-free GlnBP, it is inaccessible for initial ligand binding, since the two domains are locked up and has limited space to allow ligand to diffuse in. This is also reflected by the smallest solvent accessible surface area (SASA) of S6 (Supplementary Fig. 2). On the contrary, the open state S5 together with the two semi-closed states S3 and S4 are sampled by ligand-free GlnBP and have remarkably larger SASAs (Supplementary Fig. 2), serving as the potential conformations for initial ligand binding. The subsequent transitions have several possibilities following the kinetic network (Fig. 4a). In this scenario, “conformational selection” works for the initial selective binding to S5, S4 or S3, and “induced fit” corresponds to subsequent transitions to the closed state S6 or states that barely exist in the absence of ligand (Fig. 5). In this regard, our studies provide another case that two mechanisms are not mutually exclusive but can be collaborative for ligand binding^57,64,65^.

### Experimental examination of the conformational dynamics

**Fig. 6:**
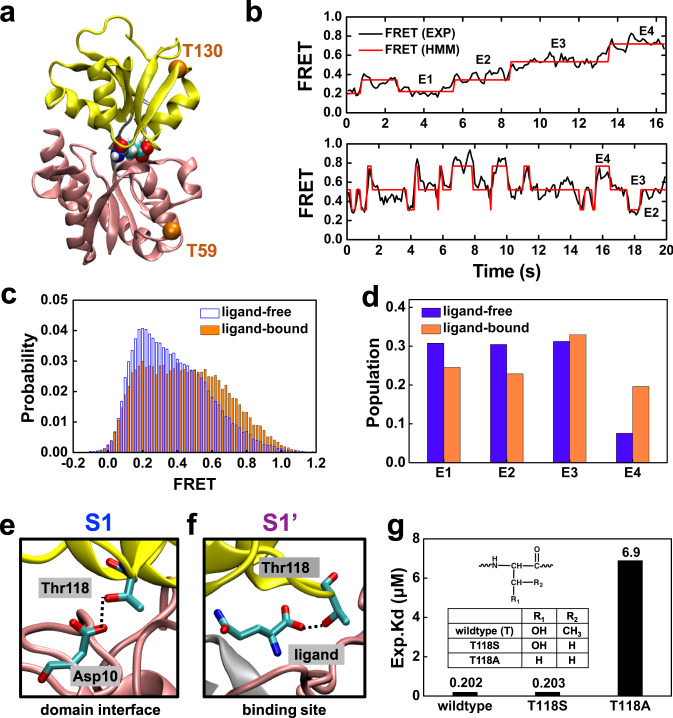
Experimental investigation of protein dynamics by smFRET and mutagenesis. **a** GlnBP structure with the orange spheres denoting the positions where fluorophores labels are attached. **b** smFRET time traces for the ligand-bound wildtype GlnBP. **c** smFRET distribution for ligand-bound (orange) and ligand-free (blue) GlnBP. **d** Populations of the four states identified in smFRET for both ligand-free and ligand-bound states. **e** In S1 state, Thr118’s side chain is interacting with Asp10 on the opposite domain. **f** In S1′ state, Thr118’s side chain forms direct interaction with the ligand. **g** Ligand-binding affinity for wildtype, T118S and T118A. The chemical structures of wildtype and mutants are shown in the inset.

One major finding in this study is ligand-bound GlnBP has multiple conformational states other than the fully closed conformation captured by the crystal structure. To examine if there exist multiple conformational states for ligand-bound GlnBP in solution, we performed smFRET experiments to measure its protein conformational dynamics (see “Methods” for details). Thr59 and Thr130 (the same residues used above for measuring the inter-domain distance in Fig. 2b) were mutated to cysteines and labeled with fluorophores (Alexa fluor 555-maleimide or Alexa fluor 647-maleimide) (Fig. 6a). We found FRET efficiency displays a broad distribution (Fig. 6b, c), suggesting the ligand-bound GlnBP can adopt other conformations other than the closed one as suggested by the crystal structure. Four states could be identified from transition density plot by hidden Markov model (HMM) state analysis (Supplementary Figs. 5–6, see “Methods” for more details). They are named as E1, E2, E3, and E4, with the efficiency values centered at 0.15, 0.31, 0.51, 0.68, respectively.

We note a quantitative comparison between the MD simulation and smFRET measurement is not feasible due to both computational limitations (e.g., not including the fluorophores in the simulation system) and many aspects that affect the experimental FRET efficiencies^66,67^. Moreover, the four states (E1 to E4) along the inter-residue distance or FRET efficiency do not correspond to the macrostates of MSM, since this single degree of freedom could not discriminate all the eight macrostates. In this regard, we only made a rough comparison using overall distribution of the inter-Cα distances between the dye-labeled residues. The calculated distance distribution can be well fitted with four Gaussian functions (Supplementary Fig. 7), centered at 4.7 Å (21%), 3.9 Å (46%), 3.6 Å (22%), and 3.4 Å (11%), the populations of which are in general agreement with the populations of the four ligand-bound states (24%, 23%, 33% and 20% for E1, E2, E3 and E4, respectively (Fig. 6d)) in smFRET measurements.

Furthermore, comparison of FRET efficiency distribution between ligand-bound and ligand-free GlnBP shows a clear population shift upon ligand binding (Fig. 6c). The state population of E4 is obviously raised while simultaneously those of E1 and E2 are reduced (Fig. 6d and Supplementary Fig. 6). One possible explanation for this population shift is that the closed S6 state shows higher ligand binding affinity as well as stronger protein–ligand interactions than the open S5 state (Fig. 3a, b), therefore promoting the corresponding population shift to the more closed conformation. An alternative interpretation is that the ligand binding has induced the appearance of four states (S1, S1′, S2, and S4′) that are not existing or rarely sampled in the absence of ligand as discussed above. Three of them adopt closed conformations, with the total populations (~24%) almost six times than that of the open one (~4%) (Fig. 2a, b). The abundance of additional closed conformations induced by ligand-binding also contributes to the population shift.

Overall, consistent with our computational model, smFRET measurements also indicate that ligand-bound GlnBP has high conformational heterogeneity in solution and ligand binding has induced the protein population shift to the more closed conformations.

Another major finding in this study is that ligand can bind in a buried pocket in the small domain. The most straightforward way to validate the small domain-binding site is to make mutations on the residues directly interacting with the ligand in these states. However, we notice these interactions are mainly through the backbone of the protein, instead of the side chain (Fig. 3c). Therefore, mutations would hardly make obvious perturbation to the binding affinity. Further structural investigations reveal that Thr118 may come into play to affect the protein’s conformational dynamics as well as the ligand binding affinity. In the S1 state, the hydroxyl group in the Thr118’s side chain forms stable hydrogen bonds (averaged distance ~3.1 Å) with the Asp10 in the opposite domain to assist locking the protein in the closed conformation (Fig. 6e). In the off-pathway state S1′, Thr118’s side chain directly interacts with the ligand’s alpha group, helping to stabilize ligand inside protein (Fig. 6f). MM-PBSA calculations estimate that Thr118 contributes ~10% to the binding free energy of S1′ state (see “Methods” for details). Based on these observations, we anticipate that Thr118 may affect conformational dynamics as well as ligand binding affinity.

Accordingly, we performed site-directed mutagenesis on Thr118 and measured the dissociation constants for the mutants (Fig. 6g, see “Methods” for details). Specifically, we designed T118S mutation to maintain the hydroxyl group in its side chain, and T118A mutation to eliminate the hydroxyl group. The results demonstrate T118S mutation has a similar binding affinity (*K*_d_ = 203 nM) to the wildtype (*K*_d_ = 202 nM). On the contrary, T118A mutation greatly decreases (~35 folds) the ligand binding affinity (*K*_d_ = 6.9 µM). These mutants underline the hydroxyl group of Thr118 is critical for ligand binding. It’s also worthy to note that Thr118’s potential function in modulating ligand binding affinity is pinpointed by the two extra conformational states from MSM. Therefore, the above experimental validation of Thr118’s effect could also serve as a proof of the multiple conformations elucidated in this work. Furthermore, it also provides direct evidence that perturbation of the conformational space can affect the binding affinity.

## Discussion

Elucidation of the conformational dynamics is essential to understanding its interplay with the biological function, especially the mechanism of molecular recognition^1–7^. “Conformational selection” and “induced fit” are two extreme models to explain the ligand binding mechanisms^8–16^. Although two mechanisms describe different dynamic processes, they both emphasize the role of protein dynamics in ligand binding. Previous X-ray crystallographic studies found GlnBP in a closed conformation upon ligand binding, and in an open conformation without ligand. The absence of ligand-free closed crystal structure leads to the assertion that GlnBP follows “induced fit” mechanism upon ligand binding, as “conformational selection” mechanism assumes protein can adopt various conformations even without the existence of ligand. However, phosphorescence spectroscopy^24^ and coarse-grained simulation^59^ have captured the inter-domain dynamics for ligand-free GlnBP. Consistently, the closed conformation was identified as one major conformation for ligand-free GlnBP using integrated computational and experimental methods^31^. These raise doubts about the commonly assumed “induced fit” mechanism for GlnBP.

In this work, we have combined MD simulation, MSM and experimental methods to examine the role of conformational dynamics in ligand binding. We have found ligand-bound GlnBP exhibits more complicated conformational dynamics than that of ligand-free GlnBP, and ligand can bind sites other than that observed in crystal structure. According to the microscopic models of conformational selection and induced fit mechanisms, the case of GlnBP can be viewed as a complex combination of the two paradigms. We also performed experiments to investigate the protein dynamics of GlnBP. Consistent with the MD and MSM results, our smFRET measurement also suggests there exist multiple conformational states for ligand-bound GlnBP. Furthermore, we identified one residue T118 that plays important role in modulating the ligand binding affinity based on our computational model and our prediction was validated by site-directed mutagenesis and binding affinity measurement. MSM constructed based on extensive MD simulations of GlnBP with the specific mutation would help to elucidated the detailed variation of protein dynamics caused by the mutation. It is noted there exists a large discrepancy between the ensemble-averaged absolute binding free energy (∆*G*_binding_) calculated using MM-PBSA method (−197.1 ± 4.1 kJ/mol) and that obtained from the ITC measurement (202 nM, corresponding to −63.0 kJ/mol at *T* = 291.15 K). One major source of this discrepancy is the conformational entropy, which is not considered in the MM-PBSA calculations^60,62^. Since the entropy’s contribution in different metastable states with the same ligand are usually similar^62^, it is reasonable to leave out the entropy term in the calculations when the relative binding affinity rather than the absolute value is needed. Actually, MM-PBSA is popular in estimating the relative binding free energies (∆(∆*G*_binding_)) in many applications with reasonable accuracy and efficiency^62,63,68^, which fits our purpose to correlate the relative ligand binding affinity of each conformational state to the protein dynamics in the current study. Altogether, these computational and experimental results suggest the complex conformational dynamics of ligand-bound GlnBP. More importantly, they provide direct evidence that binding affinity can be manipulated by regulating protein dynamics.

Complex conformational dynamics and multiple binding sites have also been found in other protein–ligand recognition systems, in sharp contrast to the simple Venus Fly-trap model. This feature might be a common paradigm of molecular recognition adopted by many proteins that have yet to be discovered. The obvious functional advantage is that the complex dynamics entails a complex kinetic network of ligand binding, which allows more flexible regulation of binding affinity. It is worth noting that multiple conformational states and their transition kinetics are very difficult to characterize experimentally. The strategy of combining experimental and computational methods shows its strength in elucidating the microscopic mechanism of molecular recognition.

## Methods

### MD simulations

Set up of MD simulation model: The ligand-bound crystal structure (PDBID: 1WDN^19^) was used as the structural basis for model construction. The terminal residues Lys4, Pro225, and Lys226 were removed to make the residue number consistent with the open crystal structure (PDBID: 1GGG^20^). The system was solvated in a dodecahedron water box with sodium chloride concentration of 0.3 mol/L. To consider the protein may transit to the open conformation, the box size was determined by the open crystal structure, making box edges at least 10 Å from the protein surface. Protein was solvated with TIP3P water molecules^69^ and counter-ions were also added to neutralize the system. AMBER03 force field parameters^70^ are used for simulating protein and ions. Partial charges of ligand were derived following RESP scheme^71,72^ and other parameters were extracted by referring to those of glutamine in the AMBER03 force field. First, 10,000-steps energy minimization was performed. Next, the system was simulated for 200 ps with position restrain on all the heavy atoms with a force constant of 10 kJ × mol^−1^ × Å^−2^ under NVT ensemble (*T* = 298 K), followed by another 500 ps position restraint under NPT ensemble (*T* = 298 K, *P* = 1 bar). Afterwards, we removed the restrain and performed a 10 ns NPT simulations, the final configuration of which was used to initiate 20 independent 100 ns production NVT (*T* = 298 K) simulations with different initial velocities. In the simulations, we applied V-rescale thermostat^73^ with the coupling time constant of 0.1 ps. A cutoff of 12 Å was used for Lennard-Jones interactions. The long-range electrostatic interactions beyond the cut-off at 12 Å were treated with the Particle-Mesh Ewald (PME) method^74^. The neighbors list was updated every 10 steps. An integration time step of 2.0 ps was used and the LINCS algorithm^75^ was applied to constrain all the bonds. We saved the snapshots every 50 ps. All the simulations were performed using Gromacs 2016.4^76,77^.

Seeding unbiased MD simulations: Several rounds of simulations were performed by seeding from different regions of the conformational space.

We collected the MD conformations from the first round of 20 × 100 ns simulations after removing the first 10 ns data from each trajectory. The conformations were divided into 20 clusters by k-center clustering algorithm^78–80^ based on the r.m.s.d. of the 220 Cα atoms after aligning the conformations by the large domain (Cα atoms of residue IDs 5 to 84 and 186 to 224). One random conformation was extracted from each cluster and used as the seed for one 100 ns MD simulation in the second round. In the same way, we collected all the conformations from the second round of 20 × 100 ns simulations and clustered them into 20 clusters. One random conformation from each cluster was selected to initiate one 100ns trajectory as the third round of MD simulations.

To examine if the ligand can reside at the domain surface when protein is open, we constructed another two models. In the first model, we aligned the closed crystal structure (PDBID: 1WDN^20^) to the open crystal structure (PDBID: 1GGG^20^) by the large-domain residues. Ligand was then extracted from the aligned closed conformation and inserted into the ligand-free-open conformation (named as “LDbind-open”). In the second model, the alignment was made according to the small-domain residues (Cα atoms of residue IDs 90 to 180) and in this way the ligand was located at the small-domain surface (named as “SDbind-open”). Similar system set up as elaborated in the subsection “Set up of MD simulation model” was used herein and 10 independent 100 ns production NVT (*T* = 298 K) simulations were performed for each model. For the “LDbind-open” model, ligand keeps binding at the large-domain surface in nine simulations. However, for the “SDbind-open” model, none of the simulations shows ligand bound at the small-domain surface and we observed ligand diffused from the small domain to the large-domain surface in two simulations. Therefore, we speculated the large-domain surface can serve as a ligand binding site when protein is in the open conformation. In this regard, we collected the conformations from the simulations with ligand binding at the protein (nine from the “LDbind-open” model and two from the “SDbind-open” model) after removing the first 10 ns from each trajectory, and did a 20-state K-center clustering and used the center conformation for seeding the second of simulations.

We combined the second and the third round of simulations from the ligand-bound closed crystal structure, as well as the second round of simulations from the two manually constructed models to do a K-center clustering and divided the conformations into 50 clusters. One random conformation was extracted from each cluster to seed the next round of simulations. In a similar way, we performed another eight rounds of MD simulations seeded from either the cluster center or one random conformation of each cluster, excepting the last two rounds in which two random conformations for each cluster were used for seeding. In total, we performed 610 × 100 ns MD simulations. In this work, we only focused on the case that ligand binds at the protein, therefore in each round of MD simulations, we examined the trajectories and removed those showing ligand dissociation before doing clustering. Finally, we have 576 MD trajectories for the MSM constructions.

### Construction and validations of MSM

The advantage of MSMs is to facilitate the investigation of long-timescale dynamics based on many short MD simulations by discretizing both conformational space and time^38–46,78,79,81–84^. Its basic idea is to partition the conformational space into metastable states, with each state corresponding to an energy minimum in the free energy landscape and the transition between them are slow. With a proper lag time ${\Delta}t$, fast motions within states are integrated out by discretizing time in units of lag time and the model becomes Markovian. That is, the probability for the system to visit a certain state at time *t* + ${\Delta}t$ is solely determined by its current position at *t*. Under the Markovian condition, the long-timescale dynamics can be obtained by propagating the transition probability matrix $T\left( \tau \right)$:

1 $$P\left( {n{\Delta}t} \right) = T({\Delta}t)^nP(0),$$

where $P\left( {n{\Delta}t} \right)$is the state population vector at time $n{\Delta}t$, and $T_{ij}$ is the element of $T\left( {{\Delta}t} \right)$, denoting the transition probability from state *i* to state *j* after a lag time of ${\Delta}t$.

We followed a commonly used “splitting and lumping” procedure to construct the MSM.

Splitting conformations into microstates with validated MSM parameters: We adopted the tICA method^44,45^ combined with the K-center algorithm^78–80^ to divide the MD conformations. The dimensionality reduction by tICA has been found to capture the slow and biological relevant conformational changes^44,45^. Optimal parameters for MSM construction were selected using a generalized matrix Rayleigh quotient (GMRQ)^43,85^ and parameters including atomsets, tIC number, tICA lag time and cluster number for K-center clustering were examined (Supplementary Fig. 8). Generally, GMRQ measures the ability of a model to capture the slowest dynamics and GMRQ scores, which are a summation over the several highest eigenvalues, serve as a good metric. The higher scores suggest the model is closer to the variational bound and is preferred. In practice, we performed 50 iterations of shuffle-split cross-validations. In each iteration, we randomly selected half number of trajectories as the training set for learning MSM and used the remaining trajectories as test set for scoring.

To consider both the inter-domain conformational change and the ligand diffusion inside the protein, we used the atomic pairwise distances between two domains, distances between the ligand and each domain, as well as the distance between the two FRET dyes inserted positions as the feature for tICA. To select the domain residues subject for the atom-pair calculations, we considered the residues within different cut off distances of the ligand. Both the ligand-bound-closed crystal structure and one representative closed conformation with ligand in the novel small-domain binding pocket were considered for the domain residue selection (Supplementary Fig. 8a–b). In the distance calculations, for the domain residues only Cα atoms were used, while for the ligand all the heavy atoms were included. In particular, we examined the cut off distances ranging from 5 to 11 Å and found GMRQ scores highest at 9 Å (Supplementary Fig. 8c). We also investigated tIC number, tICA lag times and cluster number for K-center clustering, and the parameters showing the highest GMRQ scores are selected (Supplementary Fig. 8d–f). Finally, we used 9 Å as the distance cutoff to select atom sets, 4 tICs, 10 ns as tICA lag time and divided the conformation into 750 microstates. To have better statistics within the microstates, we removed those microstates having fewer than 15 conformations (~1% of mean number of conformations per state) and got the 704 microstates for the subsequent model validation and analysis.

MSM validation: To determine the lag time ${\Delta}t$ when the system becomes Markovian, we examine the implied timescales^40,42,86^ $(\tau _k)$, which is calculated as:

2 $$\tau _k = - \tau /\ln \mu _k(\tau ),$$

where $\mu _k(\tau )$ is the *k*th eigenvalue of the transition probability matrix at lag time $\tau$. If the model is Markovian, the implied timescale $\tau _k$ becomes constant regardless of the choice of $\tau$^87^.

To take the uncertainties into account, we applied bootstrapping method by using 576 samples. For each sample, we randomly selected 576 trajectories with replacement from the ensemble of MD trajectories and calculated the implied timescales by constructing MSMs. Averaged values and errors were estimated from the 576 samples and reported in Supplementary Fig. 9a. In our model, we found the implied timescale plot reached plateau at around 30 ns, implying the system becomes Markovian after this time. Accordingly, we selected 30 ns as the lag time to construct our MSMs.

To further validate our model, we compared the residence probability (the probability for the system to remain in a certain microstate) as a function of time obtained from the propagation of the MSM with those from the original MD trajectories^41^ (Supplementary Fig. 9b) and they are in good agreement.

Examination of the convergence of conformational samplings and MSMs: In order to investigate the convergence of the MD conformational space, we compared the complete MD samplings (N rounds) with those excluding the last two rounds (N-2 rounds), and those excluding the last round (N-1 rounds) (Supplementary Fig. 1a). It’s shown that no new metastable regions in the conformational space appears as the increase of the sampling, suggesting the free energy landscape has reached reasonable convergence. We also examined the robustness of our MSMs by constructing models from the three MD datasets. The results show the implied timescales are consistent with the increment of size of MD conformational ensemble (Supplementary Fig. 1b), reflecting that our conformational sampling is sufficient to predict the dynamics of the system.

Lumping and calculations of the quantities for metastable states: For visualization and revealing the molecular mechanisms, we further lumped microstates into eight metastable states by an improved Perron-cluster cluster analysis^88,89^. To calculate the quantities for meatastable states, we used the transition probability matrix constructed at microstate level to generate 100 × 300 ms Monte Carlo trajectories. The first 100 ms was used as equilibration and removed for the calculation of quantities. The stationary populations of metastable states (Fig. 2a) and the mean first passage time (Fig. 4a) for each transition were calculated by averaging the values over the 100 trajectories.

### Estimation of binding free energies

We applied MM-PBSA method^60,61^ to calculate the binding free energies for each metastable state (Fig. 3a). We randomly selected 10 conformations from each microstate for the MM-PBSA calculations with SASA model for computing non-polar solvation energy. Default parameters are used for the calculations and the temperature is set to 298 K (MD simulation temperature). We performed 1000 trials to estimate the average and standard deviations for the binding free energy for each metastable state. In each trial, we randomly selected one conformation from each microstate and calculated its binding free energy; the binding free energy of one metastable state is then calculated as the weighted sum over those of all the microstates belonging to the corresponding metastable state, and the weight is determined by the relative population of the microstate in the respective metastable state.

The MM-PBSA method was capable to estimate the individual residue’s contribution to the overall binding energy. The averaged binding energy from T118A was derived in a similar way as illustrated above.

The ensemble-averaged binding free energy contributed by all metastable states was computed as the weighted sum of the binding free energy of each microstate. The weight is determined by the population of the respective microstate. Mean and standard deviation were estimated by performing 1000 iterations of such calculations.

### Protein preparation

*Escherichia coli* glnH was cloned from genomic DNA of *Escherichia coli* BL21(DE3) strain by standard PCR and subcloned into a pET28a vector between *Eco*R I and *Sal* I. The plasmid was amplified in DH5α and transformed into BL21(DE3) strain. C-terminal His-tagged GlnBP was expressed in *E. coli* BL21(DE3) in LB medium at 37 °C overnight with the induction of IPTG and purified by Ni^2+^-NTA agarose affinity chromatography. GuHCl was then added into GlnBP solution to a final concentration of 6 M to fully denature the protein and detach possibly existing L-glutamine. The mixture was purified by Superdex 75 size-exclusion chromatography column (GE Healthcare) to refold the protein and keep the purified ligand-free GlnBP in PBS buffer for further usage. In order to study the dynamics property of ligand-bound GlnBP, excess amount of L-glutamine was added into ligand-free GlnBP solution before each experiment.

### Site-directed mutagenesis

The mutations of GlnBP in Thr118 were sequentially introduced by PCR reactions based on the plasmid *glnH*-pET28a as mentioned in the protein preparation part. In the first step, the DNA fragment of 5′-end to the mutation site and the fragment of mutation site to the 3′-end were synthesized by PCR separately. In the second step, the whole mutated *glnH* DNA was synthesized with the above two fragments as template. The mutated *glnH* DNA was then subcloned into the pET28a vector for plasmid amplification and protein purification. All plasmids were confirmed by sequencing.

### Isothermal titration calorimetry

ITC measurements were performed on an ITC200 Micro calorimeter (MicroCal) at 18 °C. All samples were dissolved in 50 mM PBS buffer. The titrations were carried out by injecting 40-μl aliquots of the ligand (0.5 and 1 mM ligand have used for GlnBP wild type and mutations, respectively) into protein (0.05 mM GlnBP wild type and 0.1 mM GlnBP mutations, respectively) at time intervals of 2 min to ensure that the titration peak returned to the baseline. The titration data were analyzed using the program Origin7.0 and fitted with the one-site binding model.

### smFRET experiment

The threonine 59 and threonine 130 of GlnBP were mutated to cysteines by standard PCR protocol. 50 μM GlnBP-T59C/T130C was labeled by 200 μM FRET donor (Alexa fluor 555-maleimide, Thermo Fisher Scientific Inc., MA, U.S.) and 400 μM FRET acceptor (Alexa fluor 647-maleimide, Thermo Fisher Scientific Inc., MA, U.S.) by following the vendor provided protocol. The unreacted dye was separated from the labeled protein by using size-exclusion chromatography (SEC).

In order for immobilization of fluorescence-dye-labeled protein to carry out FRET experiment, the glass coverslip and drilled glass slide were cleaned by sonicating in water and ethanol three times respectively followed by being etched in plasma cleaner (PDC-002, Harrick Plasma Inc., NY, U.S.) for 5 min to destroy the residual dusts. The coverslip was stuck on the bottom of the drilled glass slide to make a flow cell. 100 μl 0.1 mg/ml poly-lysine-PEG-NTA (PLL(20)-g[3.5]-PEG(2)-NTA, SuSoS AG Inc., Switzerland) solution was added to the flow cell and incubated for 20 min in order to create a layer of PEG on the coverslip surface and passivate it. The flow cell was washed with buffer thoroughly and incubated with 0.1 M NiCl_2_ solution to introduce Ni^2+^ to the NTA. After 20 min incubation and complete wash, 1 nM fluorescence-dye-labeled GlnBP solution (50 mM phosphate buffer, pH 6.8) was added to the flow cell in order to tether the protein down to the glass surface upon the binding between the His-tag of GlnBP and the NTA group on the PEG layer.

The single molecule FRET images were taken by using a home-built wide-field fluorescence imaging system with an exposure time of 100 ms. The 532 nm laser beam was focused by a wide field 300 mm convex lens and then coupled to the microscope (IX-73, Olympus, Japan) through the back port. Eventually the beam illuminated a circular area on the coverslip and excited the labeled protein molecules in this area. The fluorescence of donor and acceptor was split by a dual image splitter (Optosplit II, Andor Technology Plc., U.K.) and the separated fluorescent images were detected by different areas of an EMCCD camera (iXon 897 Ultra, Andor Technology Plc., U.K.). The acceptor itself was excited at 632 nm in the control measurement.

The software iSMS^90^ was used to extract and calculate the traces of fluorescence intensity of donor and acceptor from consecutively fluorescent images. We determined the number of FRET efficiency states by HMM analysis using vbFRET software^91^ (Fig. 6b). The change points of transitions between states in ideal HMM trajectories of ligand-bound GlnBP were used to build the transition density plot and it showed major transitions between four FRET efficiency states. A threshold analysis was performed to obtain the population of each state in both ligand-free and ligand-bound GlnBP systems. First, each point on TDP of ligand-bound GlnBP was dressed with a 2D normalized Gaussian function to facilitate its construction^92^. Then the constructed TDP was fitted by six 2D Gaussian functions to obtain the center and width of peaks. Finally, the population of each state in both ligand-free and ligand-bound GlnBP systems was obtained by using the threshold which set by the fullwidth half-height of the Gaussian distribution (Supplementary Fig. 5). These thresholds were ideal FRET 0.080−0.250 for E1, 0.255−0.415 for E2, 0.435−0.625 for E3 and 0.630–0.790 for E4. Histograms of FRET were obtained by combining all the time points of raw FRET trajectories corresponding to each state and fitted to the four Gaussian functions (Supplementary Fig. 6).

### Statistics and reproducibility

Unless stated otherwise, the means and standard deviations for the quantities of each state were calculated based on all the MD conformations belonging to the specific state.

### Reporting summary

Further information on research design is available in the Nature Research Reporting Summary linked to this article.

## Supplementary information

Supplementary Information

Description of Additional Supplementary Files

Supplementary Data 1

Reporting Summary

Peer Review File

## Data Availability

All data generated and analyzed during this study are included in the article or from the corresponding author on request. Source data underlying plots shown in figures are provided in Supplementary Data 1.

## Code availability

MSMbuilder3.8 was used for the MSM construction.
