# Supplementary material for: Ligand-bound glutamine binding protein assumes multiple metastable binding sites with different binding affinities
Source: Commun Biol. 2020 Aug 3;3:419. doi: 10.1038/s42003-020-01149-z (PMC7400645; doi:10.1038/s42003-020-01149-z)
Supplement: Supplementary file 1 — Supplementary Information [file 42003_2020_1149_MOESM1_ESM.pdf]

Supplementary Figures

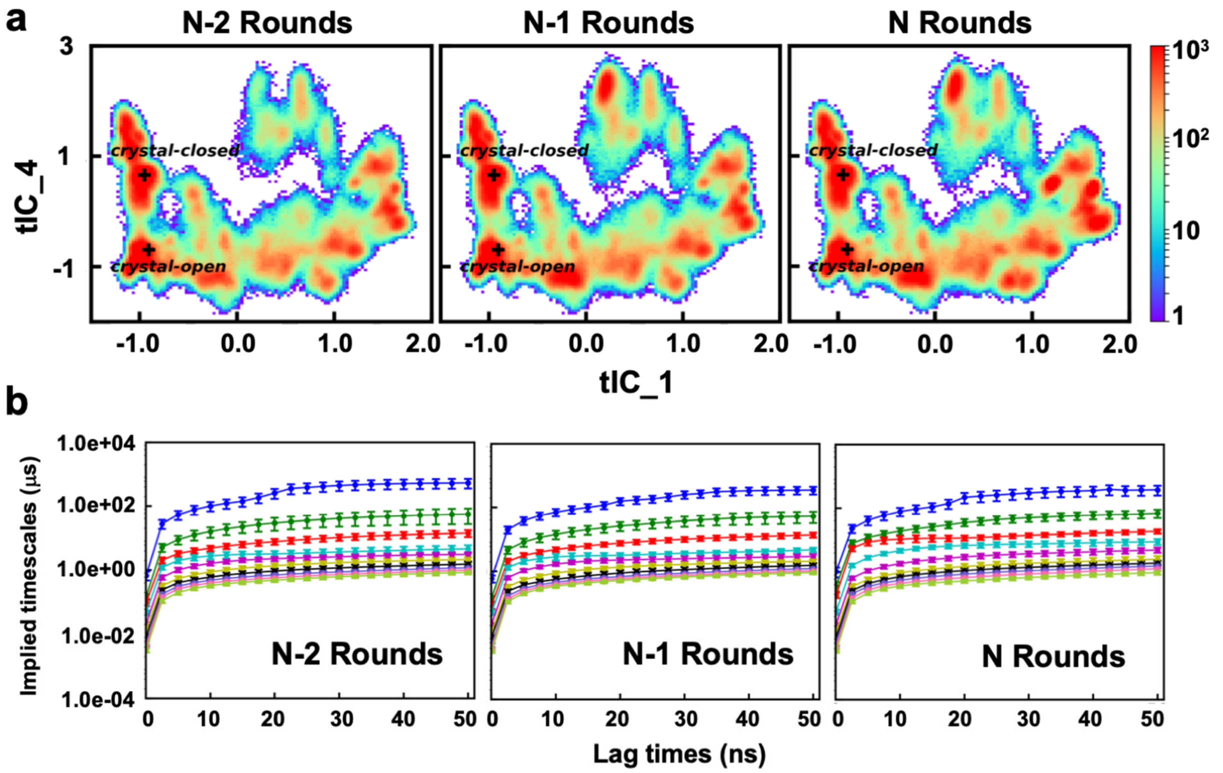

Supplementary Fig. 1 Validation of the convergence of MD conformational space and MSMs. a. Projection of MD conformations onto the 1st and 4th tICs with the increase of rounds of simulations. **b.** Implied timescales as a function of lag time predicted by MSMs constructed based on different MD datasets as used in **a**. The MSMs constructed based on N-2 rounds and N-1 rounds of simulations contain 700 and 750 microstates, respectively. The errors of the implied timescales were estimated from bootstrapped samples, the number of which are the same as the total number of trajectories for the corresponding MD dataset. For each sample, the same number of trajectories were randomly selected with replacement from the ensemble of MD trajectories in use.

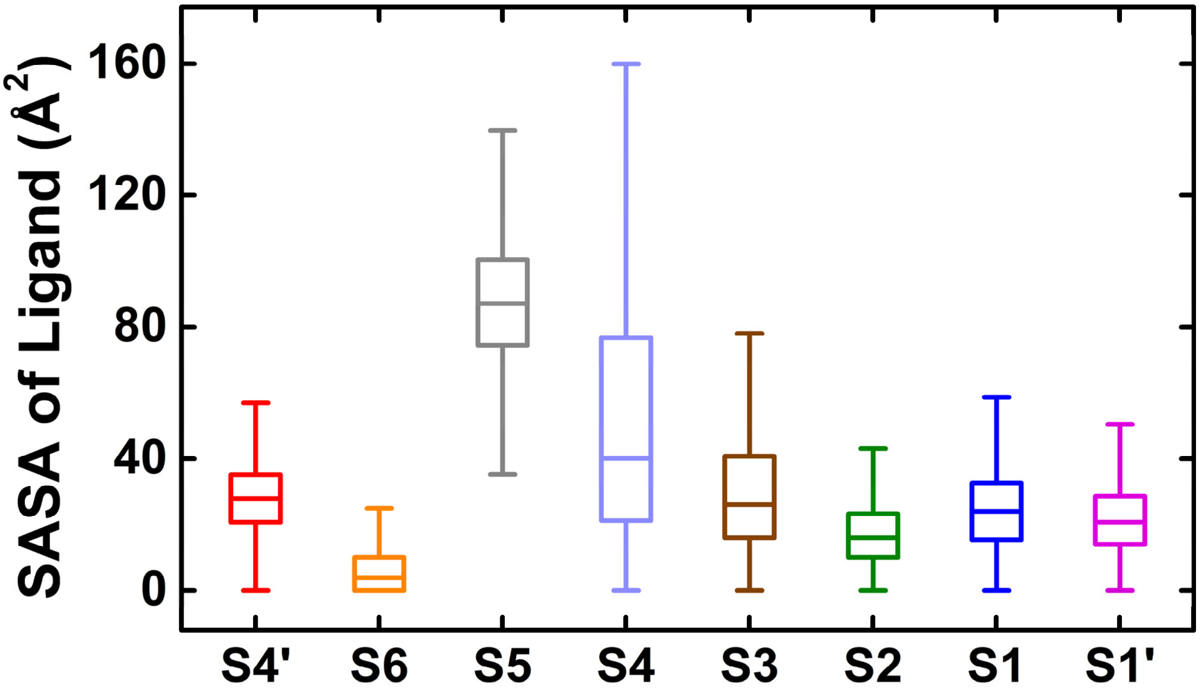

Supplementary Fig. 2 Solvent accessible surface area of ligand in the macrostates. Box plot for each macrostate was calculated using all the MD conformations belonging to the specific state.

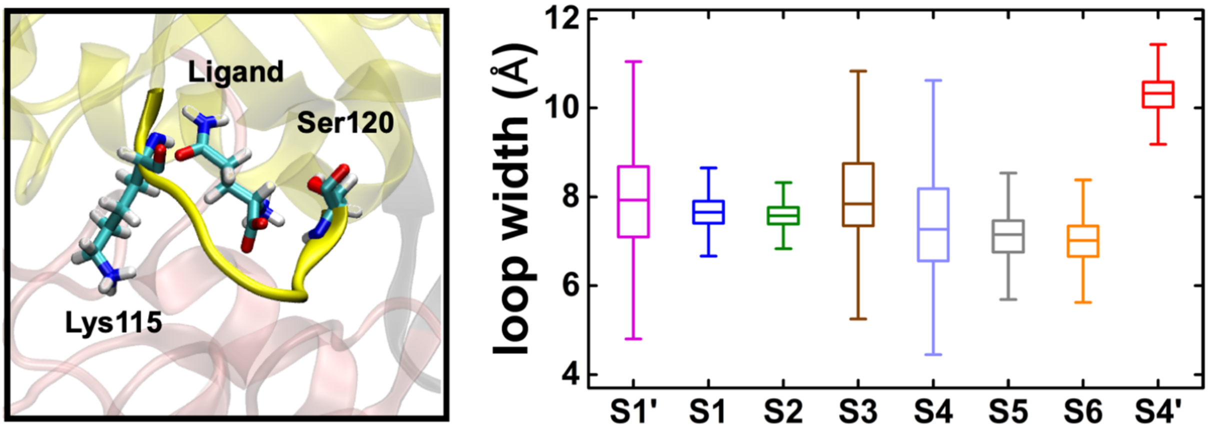

Supplementary Fig. 3 One loop between the strand J and helix IV expands in state S4'. The loop width for each macrostate was measured using the distance between the Cα atom of Lys115 and Ser120, which are at the two ends of the loop. All the MD conformations belonging to the specific state was included in the statistics of loop width for each macrostate.

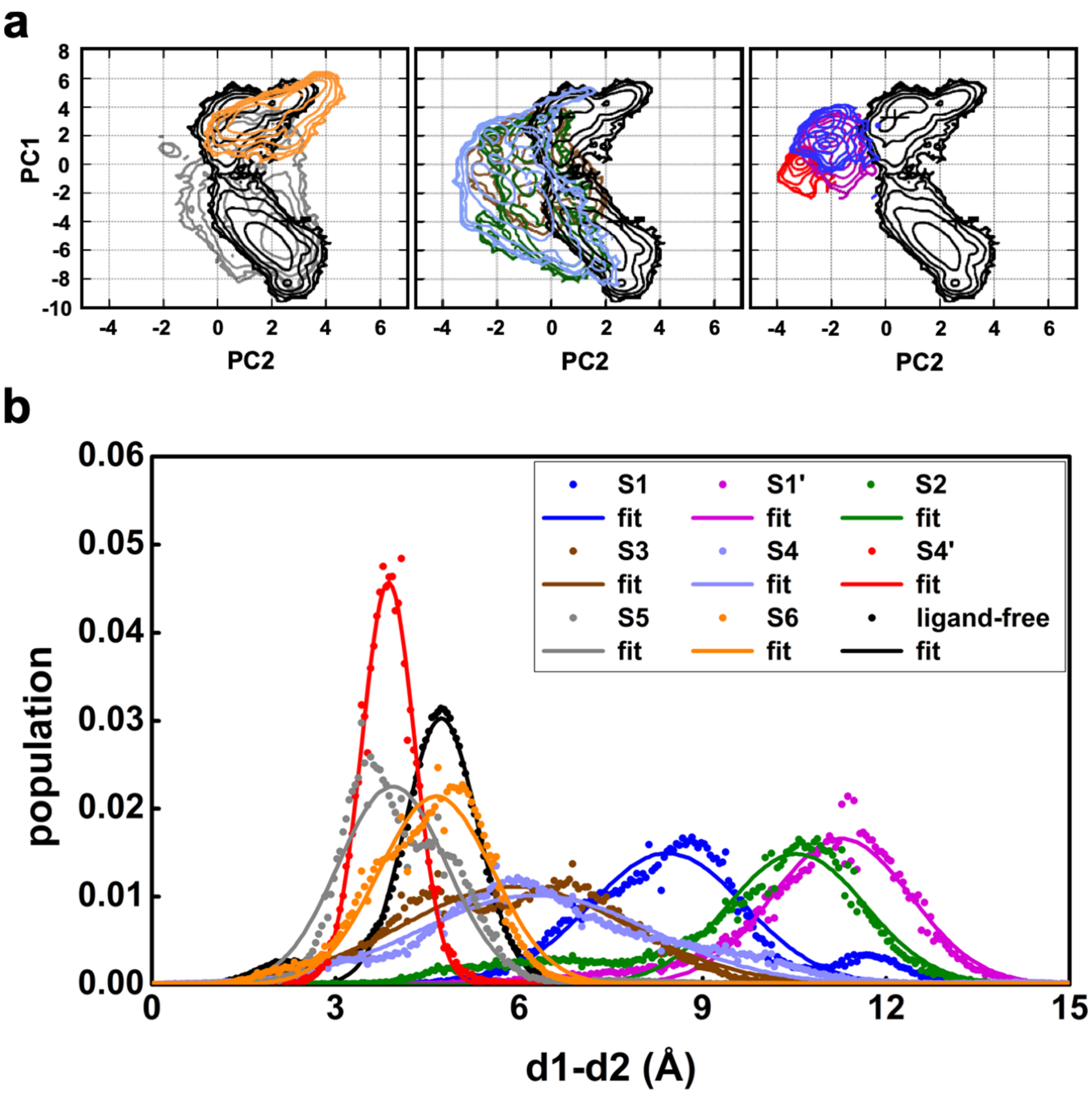

Supplementary Fig. 4 Comparison between the conformational space of ligand-bound GlnBP and that of ligand-free GlnBP. a. Projection of each metastable state onto the top two principal components, in comparison with that of ligand-free GlnBP (in black). The color code for each metastable state is the same as that used in Fig. 2a. Principal component analysis was performed based on all Cα atoms using all the conformations of ligand-bound GlnBP. **b.** Distribution of the distance difference between d1 and d2 (Fig. 4b) for ligand-free GlnBP conformations, and each metastable state of ligand-bound GlnBP (in filled circles). All the MD conformations falling into the specific state were included in the calculation of the distance distribution. Gaussian function was used to fit the distribution and the fitted curve was shown in solid line.

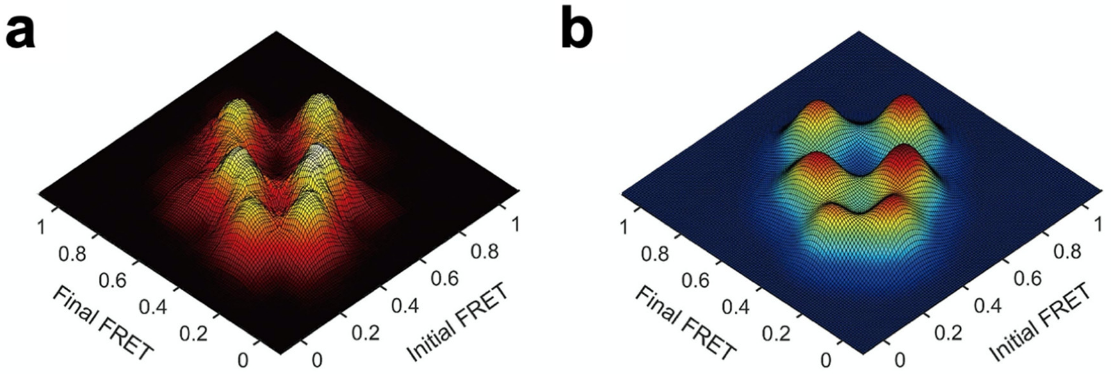

Supplementary Fig. 5 Transitions between multiple FRET states of *wild type* holo-GlnBP. a. Transition density plot (TDP) constructed from the experimental data using HMM analysis results. **b.** 2D-Gaussian fitting of TDP. R square of fitting is 0.96.

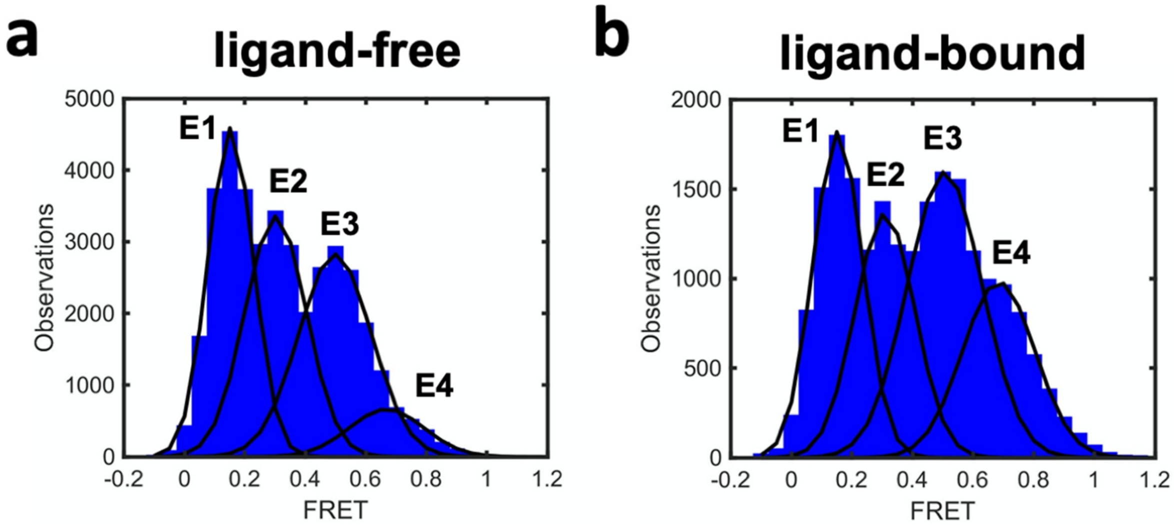

Supplementary Fig. 6 Histograms of the FRET efficiencies for the four states identified using HMM analysis in the ligand-free and ligand-bound GlnBP.

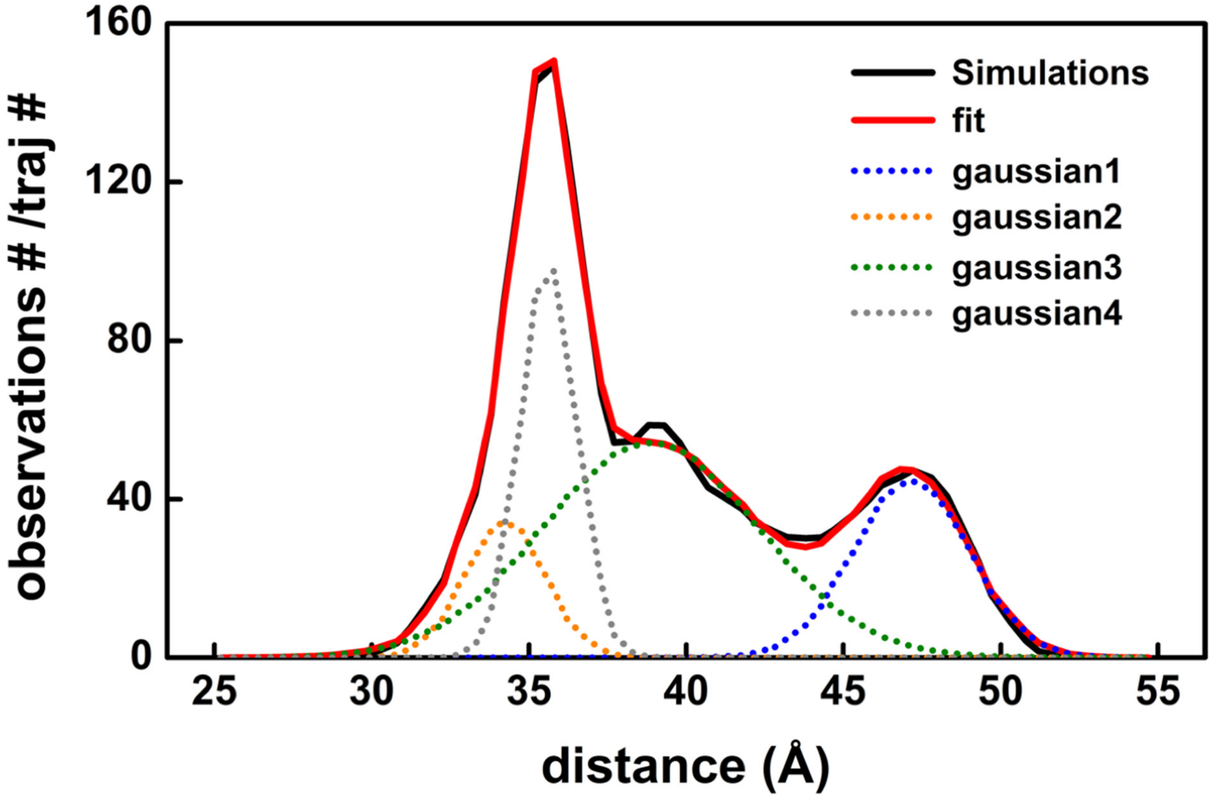

	gaussian1	gaussian2	gaussian3	gaussian4
Peak position (Å)	47.1	34.2	38.9	35.6

Supplementary Fig. 7 Distribution of distance between the two Cα atoms at dyes inserted positions. The solid black lines are the distance distribution calculated based on the MD conformational ensemble and the solid red line is the fitting curve using a summation of four Gaussian functions. The four dashed lines are the four Gaussian curves used for the fitting and the table shows the peak position, with an individual color for each Gaussian function.

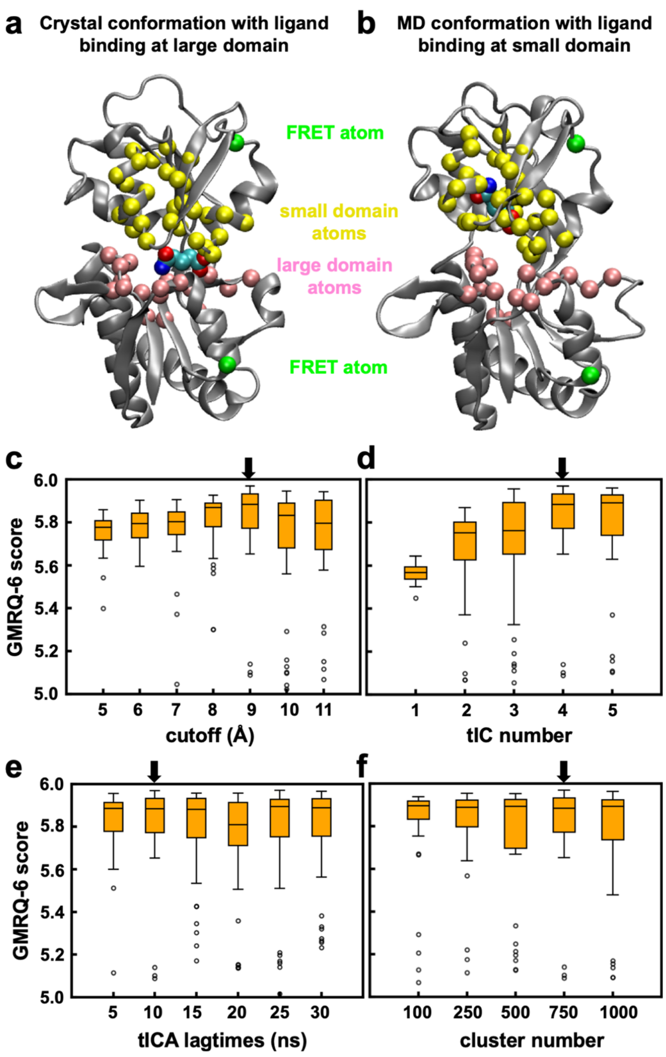

Supplementary Fig. 8 Validation of parameters to construct MSM. a-b. Atoms used for tICA atom-pair featurization include Cα atoms in large domain (in yellow balls) and small domain (in pink balls), heavy atoms of the ligand, as well as the two Cα atoms in the locations where the FRET labels are inserted. **a** shows the crystal structure when ligand is at the large domain and **b** shows one MD conformation with ligand at the small domain. **c-f.** Box plot of GMRQ test over parameter for MSM construction, with **c** for the the distance cutoff to select the atomsets, **d** for the tIC number, **e** for the tICA lagtimes and **f** for the the cluster number of K-center clustering. The optimal parameters are denoted with the black arrows. In **c**-**f**, statistics were estimated by 50 iterations of shuffle-split cross-validations. In each iteration, we randomly selected half number of trajectories as the training set for learning MSM and the remaining trajectories were used as test set for scoring.

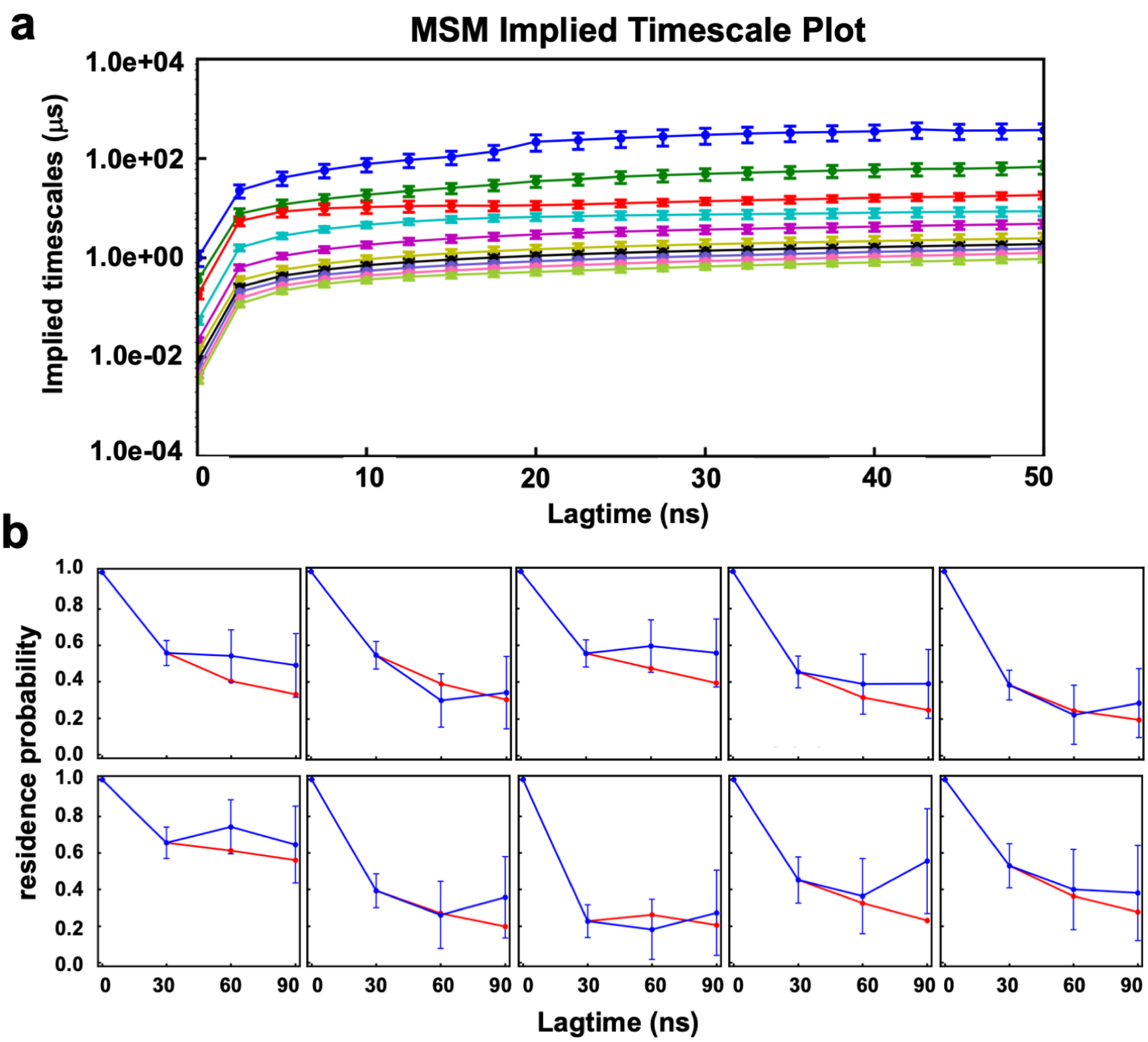

Supplementary Fig. 9 MSM validation. a. Implied timescale plot of MSM. The means and standard deviations were estimated by bootstrapping method using 576 samples. For each sample, we randomly selected 576 trajectories with replacement from the ensemble of MD trajectories and calculated the implied timescales. **b.** Validation of Markovian properties for the top 10 populated microstates. Residence probability calculated using MSM (red) is in comparison with that computed directly from MD simulations (blue). The means and standard deviations of MD residence probability for one microstate at a given lag time were estimated by the corresponding element in the transition count matrix constructed at the specific lag time using all the 576 trajectories.
