# Supplementary material for: Ligand-bound glutamine binding protein assumes multiple metastable binding sites with different binding affinities
Source: Commun Biol. 2020 Aug 3;3:419. doi: 10.1038/s42003-020-01149-z (PMC7400645; doi:10.1038/s42003-020-01149-z)
Supplement: Supplementary file 2 — Description of Additional Supplementary Files [file 42003_2020_1149_MOESM2_ESM.pdf]

Description of Additional Supplementary Files

File Name: **Supplementary Data 1**
Description: Source data for main text figures
