# Supplementary material for: Ligand-bound glutamine binding protein assumes multiple metastable binding sites with different binding affinities
Source: Commun Biol. 2020 Aug 3;3:419. doi: 10.1038/s42003-020-01149-z (PMC7400645; doi:10.1038/s42003-020-01149-z)
Supplement: Supplementary file 4 — Reporting Summary [file 42003_2020_1149_MOESM4_ESM.pdf]

nature research

Corresponding author(s): Lu Zhang, Wenning Wang

Last updated by author(s): Jun 16, 2020

Reporting Summary

Nature Research wishes to improve the reproducibility of the work that we publish. This form provides structure for consistency and transparency in reporting. For further information on Nature Research policies, see our Editorial Policies and the Editorial Policy Checklist.

Statistics

For all statistical analyses, confirm that the following items are present in the figure legend, table legend, main text, or Methods section.

n/a	Confirmed	
☒	☐	The exact sample size (*n*) for each experimental group/condition, given as a discrete number and unit of measurement
☒	☐	A statement on whether measurements were taken from distinct samples or whether the same sample was measured repeatedly
☒	☐	The statistical test(s) used AND whether they are one- or two-sided<br>*Only common tests should be described solely by name; describe more complex techniques in the Methods section.*
☒	☐	A description of all covariates tested
☒	☐	A description of any assumptions or corrections, such as tests of normality and adjustment for multiple comparisons
☐	☒	A full description of the statistical parameters including central tendency (e.g. means) or other basic estimates (e.g. regression coefficient) AND variation (e.g. standard deviation) or associated estimates of uncertainty (e.g. confidence intervals)
☒	☐	For null hypothesis testing, the test statistic (e.g. *F*, *t*, *r*) with confidence intervals, effect sizes, degrees of freedom and *P* value noted<br>*Give P values as exact values whenever suitable.*
☒	☐	For Bayesian analysis, information on the choice of priors and Markov chain Monte Carlo settings
☒	☐	For hierarchical and complex designs, identification of the appropriate level for tests and full reporting of outcomes
☒	☐	Estimates of effect sizes (e.g. Cohen's *d*, Pearson's *r*), indicating how they were calculated

Our web collection on statistics for biologists contains articles on many of the points above.

Software and code

Policy information about availability of computer code

Data collection	MD simulations were performed using gromacs2016.4.
Data analysis	MSM analysis was performed using msmbuilder3.8.

For manuscripts utilizing custom algorithms or software that are central to the research but not yet described in published literature, software must be made available to editors and reviewers. We strongly encourage code deposition in a community repository (e.g. GitHub). See the Nature Research guidelines for submitting code & software for further information.

Data

Policy information about availability of data

All manuscripts must include a data availability statement. This statement should provide the following information, where applicable:

- Accession codes, unique identifiers, or web links for publicly available datasets
- A list of figures that have associated raw data
- A description of any restrictions on data availability

All data generated and analyzed during this study are included in the article and the supplementary information or from the corresponding author on request. Raw data for Fig. 3b, 3c, 4c, 6b, 6c, 6d and 6g were combined in one Excel file and uploaded as the "Source Data".

Field-specific reporting

Please select the one below that is the best fit for your research. If you are not sure, read the appropriate sections before making your selection.

☒ Life sciences ☐ Behavioural & social sciences ☐ Ecological, evolutionary & environmental sciences

For a reference copy of the document with all sections, see nature.com/documents/nr-reporting-summary-flat.pdf

Life sciences study design

All studies must disclose on these points even when the disclosure is negative.

Sample size	Computational Part:<br>Details about the sample size for the MD simulations can be found in the "Methods" section. The convergence of the sample size for constructing Markov State Model is validated by the conformational space and the implied timescales, which is shown in Supplementary Figure 1 and discussed in the "Methods" section.<br><br>Experimental Part:<br>Sample sizes were not computed as part of the study design. Results from smFRET experiments were compared with previous experiments of apo-GlnBP. For all experiments of GlnBP mutants, we included control experiments of wild type protein.
Data exclusions	No data was excluded
Replication	The ITC titration and smFRET experiment were carefully controlled and comparison between WT and mutants, ligand free and ligand-bound samples were presented.
Randomization	Not applicable.
Blinding	Group allocation considerations are not applicable to our smFRET experiment and computational work using Markov State Model based on MD simulations.

Reporting for specific materials, systems and methods

We require information from authors about some types of materials, experimental systems and methods used in many studies. Here, indicate whether each material, system or method listed is relevant to your study. If you are not sure if a list item applies to your research, read the appropriate section before selecting a response.

Materials & experimental systems

n/a	Involved in the study	
☒	☐	Antibodies
☒	☐	Eukaryotic cell lines
☒	☐	Palaeontology and archaeology
☒	☐	Animals and other organisms
☒	☐	Human research participants
☒	☐	Clinical data
☒	☐	Dual use research of concern

Methods

n/a	Involved in the study	
☒	☐	ChIP-seq
☒	☐	Flow cytometry
☒	☐	MRI-based neuroimaging
