# Supplementary material for: Ligand-bound glutamine binding protein assumes multiple metastable binding sites with different binding affinities
Source: Commun Biol. 2020 Aug 3;3:419. doi: 10.1038/s42003-020-01149-z (PMC7400645; doi:10.1038/s42003-020-01149-z)
Supplement: Supplementary file 5 — Peer Review File [file 42003_2020_1149_MOESM5_ESM.pdf]

Reviewers' comments:

Reviewer #1 (Remarks to the Author):

This is an article to illustrate the relationships between protein conformational dynamics and biological functions, especially protein-ligand interactions by integrating molecular dynamics simulations, Markov state model analysis and experiments. This study shows that ligand-bound GlnBP with diverse conformations has different binding affinity and and demonstrated the ligand binding mechanisms. This study is interesting and will provide useful evidences for studying protein functions. However, the authors need to clarify several concerns.
(1) In page 7, 148-152 lines, the author mentioned that ".... binding pocket in the small domain is relatively buried and therefore that is not sensitive to the protein's opening-closing motion." Is there any evidence to show the correlation between domain size and the protein's opening-closing motion?
(2) In page 8, line 160-161, "... we found the ligand can bind at different sites with
161 diverse affinities when protein shows similar opening extent ..." Can you give some examples on how diverse the binding affinities and how similar the protein showing opening extent ?
(3) In page 9, line 188-189, ".... structural rearrangements involving helix V (residues 138-146), helix VI (residues 160-168) and strand J (residues 111-115) in the small domain.." How do you define a small domain and a big domain? Is there any criteria ?
(4) In page 13, "...The most straightforward way to validate the small domain-binding site is to make mutations on the residues directly interacting with the ligand in these states ..." Thr118 may affect protein conformational dynamics and the ligand binding affinity. If Thr118 is mutated the binding affinity will be changed or not ? How about protein conformational dynamics?

Reviewer #2 (Remarks to the Author):

Here the authors combine MD simulations, Markov state model (MSM) analysis and experiments to obtain insight into the conformational dynamics of ligand-bound glutamine binding protein (GlnBP). Their integrated analysis indicates that ligand-bound GlnBP is highly flexible with multiple metastable binding sites. Interestingly, these present a more complex energy landscape than the one in the absence of ligand. The conformational ensemble of GlnBP presents different binding affinities and of note, complex transition kinetics, suggesting a concerted ligand binding mechanism. The authors then carry out single molecule FRET and mutagenesis experiments. These confirm their MSM-derived conformational ensemble and their binding mechanism.

I like this paper since it is not only comprehensive, meticulous and very carefully done; in particular, it elucidates the linkage between protein dynamics and ligand binding, clearly showing that protein dynamics is essential for ligand binding. They also propose a complex regulatory network that may underlie the apparent binding affinity.

This is fundamental research into the basic science underlying protein conformational behavior, its dynamics and function. The paper is very carefully prepared and is very clear. Readers should find it of interest.

Reviewer #3 (Remarks to the Author):

The authors present a computational study of the glutamine binding protein, and through a Markov State Model for the ligand-bound and unboud states propose a scheme for a ligand binding scheme that is coupled to internal protein dynamics. The study is complemented by FRET and mutagenesis

experiments. Although in principle interesting, the study suffers from a major flaw based on which I cannot recommend it for publication.

The ligand binding affinity for each state is estimated based on a Poisson-Boltzmann surface area (MM-PBSA) method. Although the experimental affinity for the ligand is reported in the high nanomolar range (which corresponds to about 40 kJ/mol), the computationally reported affinities are up to > 300 kJ/mol (Fig. 3). This large discrepancy for the computational affinity precludes any mechanistic interpretation or comparison to experiment.

Response to Reviewer#1's Comments:

This is an article to illustrate the relationships between protein conformational dynamics and biological functions, especially protein-ligand interactions by integrating molecular dynamics simulations, Markov state model analysis and experiments. This study shows that ligand-bound GlnBP with diverse conformations has different binding affinity and and demonstrated the ligand binding mechanisms. This study is interesting and will provide useful evidences for studying protein functions. However, the authors need to clarify several concerns.

Reply: We thank the reviewer for his/her carefully reading of our manuscript. This reviewer thinks our study is interesting and "*provide useful evidences for studying protein functions*" However, he/she also made some comments for us to improve our manuscript. In this revision, we have fully addressed his/her comments and following is our point-to-point response to these comments.

1. In page 7, 148-152 lines, the author mentioned that ".... binding pocket in the small domain is relatively buried and therefore that is not sensitive to the protein's opening-closing motion." Is there any evidence to show the correlation between domain size and the protein's opening-closing motion?

Reply: We would like to thank the reviewer for this insightful comment. We have evidence to show that the small domain binding pockets in S1 and S2 states are relatively buried, and ligand only interacts with protein residues in the small domain, which render the binding affinity in these two states not sensitive to the opening-closing motion.

We used solvent accessible surface area (SASA) to estimate the exposure extent of the ligand binding pockets. As shown in Supplementary Fig. 4, both S1 and S2 states show small SASAs (~25 $Å^2$ for S1 and ~18 $Å^2$ for S2), suggesting the binding pockets are buried. Furthermore, ligand in S1 and S2 states only interacts with the small domain residues (Fig. 3c) and does not form interaction with large domain residues (Fig. 3b). Therefore, although S1 and S2 demonstrate different protein conformations (Fig. 2b), the opening-closing motions would not perturb the ligand-protein interactions in S1 and S2 states. These observations altogether explain why S1 and S2 have similar ligand binding affinity (Fig. 3a). even though they demonstrate different protein conformations (Fig. 2b).

To make this point clearer to the readers, we have changed the sentence in page 7 as follows:

from "*This can be explained by the fact that binding pocket in the small domain is relatively buried and therefore that is not sensitive to the protein's opening-closing motion. This also matches our evaluation that S1 and S2 states demonstrate similar interactions between ligand and the small domain residues Ala113, Lys115, Gly119, Leu155 and Ser120 (Fig. 3c).*"

to "*This can be explained by the observation that their binding pockets in the small domain are relatively buried (Supplementary Fig. 4) and ligand only interacts with protein residues in the small domain in these two states. Specifically, ligand in S1 and S2 states shows similar interactions with small domain residues Ala113, Lys115, Gly119, Leu155 and Ser120 (Fig. 3c), while does not interact with the large domain residues (Fig. 3b). Thus, the opening-closing motion would not perturb the ligand-protein interactions in S1 and S2 states. These observations altogether explain why S1 and S2 have similar ligand binding affinity (Fig. 3a). even though they demonstrate different protein conformations (Fig. 2b).*

2. In page 8, line 160-161, "... we found the ligand can bind at different sites with diverse affinities when protein shows similar opening extent ..." Can you give some examples on how diverse the binding affinities and how similar the protein showing opening extent ?

Reply: We thank the reviewer for this good suggestion. The example can be presented by comparing the binding affinity and opening extent of S5 state with those of S2 state. The extent of opening was measured by the d_{FRET}, *i. e.* Cα-Cα distance between Thr59 (large domain) and Thr130 (small domain). As shown in Fig. 2b, S2 state shows d_{FRET} of 43.1±3.9Å, resembling 46.1±3.2Å for S5 states, suggesting that their opening extents are similar. However, the binding affinities of S2 and S5 are significantly different, with the binding affinity of S5 state obviously higher than that of S2 state (Fig. 3a). Based on these observations, we proposed that even though conformational states show similar opening extent, different binding sites can give rise to diverse binding affinities.

As suggested by the reviewer, we have added the above discussions in page 7 of the current manuscript and accordingly changed the sentence as follows:

from "*Based on these observations, we found the ligand can bind at different sites with diverse affinities when protein shows similar opening extent ...*"

to "*Furthermore, we found different binding sites can give rise to diverse binding affinities when protein shows similar opening extent. As shown in Fig. 2b, S2 and S5 states show dFRET of 43.1±3.9Å and 46.1±3.2Å, respectively, suggesting that their opening extent are similar. However, the binding affinities of S2 and S5 are significantly different, with the binding affinity of S5 state obviously higher than that of S2 state (Fig. 3a). ...*"

3. In page 9, line 188-189, ".... structural rearrangements involving helix V (residues 138-146), helix VI (residues 160-168) and strand J (residues 111-115) in the small domain.." How do you define a small domain and a big domain? Is there any criteria ?

Reply: We would like to thank the reviewer for pointing this out. As shown in Fig.1, the structural topology of GlnBP is formed by two globular domains linked through peptide hinges. The large domain contains both the N terminal and C terminal residues (protein residues 5 to 84 and 186 to 224), the small domain contains protein residues 90 to 180, and the hinge region contains protein residues 85 to 89 and 181 to 185. The terms "large domain" and "small domain" have been used in the paper (*J. Mol. Biol.* 262, 225-242 (1996)) that reported the crystal structure of GlnBP in the ligand-bound state, as well as papers investigating GlnBP from other groups (for example, *Biophys. J.* 92, 1326-1335 (2007), *Proteins* 71, 743-750 (2008), etc.). The "large" and "small" terms are used to differentiate the number of protein residues in each domain.

We have added the definition of large domain and small domain in page 3 of the current manuscript by changing the sentence as follows:

from "*..., GlnBP is composed of two globular domains connected by a hinge region, ...*"

to "*…, GlnBP is composed of two globular domains, the large domain (protein residues 5 to 84 and 186 to 224) and the small domain (protein residues 90 to 180), connected by a hinge region (protein residues 85 to 89 and 181 and 185), ...*"

4. In page 13, "...The most straightforward way to validate the small domain-binding

site is to make mutations on the residues directly interacting with the ligand in these states ..." Thr118 may affect protein conformational dynamics and the ligand binding affinity. (1) If Thr118 is mutated the binding affinity will be changed or not ? (2) How about protein conformational dynamics?

Reply to point (1): We performed site-directed mutagenesis on Thr118 and measured the ligand dissociation constants for the mutants. Specifically, we designed T118S mutation to maintain the hydroxyl group in its side chain, and T118A mutation to eliminate the hydroxyl group. The results demonstrate that the T118S mutation has a similar binding affinity (K_d=203 nM) to the wildtype (K_d=202 nM). On the contrary, T118A mutation, greatly decreases (~35 folds) the ligand binding affinity (K_d=6.9 μM). These mutants underline the hydroxyl group of Thr118 is critical for ligand binding.

To clarify these results to readers, we have changed the sentence in page 15 as follows:

from "*..., we performed site-directed mutagenesis on Thr118 and measured the dissociation constants for T118S and T118A mutants (Fig. 6g, see Methods and Supplementary Note 4 for details). The result demonstrates T118S mutation, which maintains the hydroxyl group in the side chain, has a similar binding affinity (K_d=203nM) to the wildtype (K_d=202 nM). On the contrary, T118A mutation, which eliminates the hydroxyl group, greatly decreases (~35 folds) the ligand binding affinity (K_d=6.9 μM).*"

to "*..., we performed site-directed mutagenesis on Thr118 and measured the dissociation constants for the mutants (Fig. 6g, see Methods and Supplementary Note 4 for details). Specifically, we designed T118S mutation to maintain the hydroxyl group in its side chain, and T118A mutation to eliminate the hydroxyl group. The results demonstrate T118S mutation has a similar binding affinity (K_d=203nM) to the wildtype (K_d=202nM). On the contrary, T118A mutation greatly decreases (~35 folds) the ligand binding affinity (K_d=6.9μM).*"

Reply to point (2): Because the hydroxyl group Thr118 in the small domain is interacting with the Asp10 in the large domain in S1 state and the hydroxyl group of Thr118 is interacting with the ligand in S1' state (Fig. 6e), we anticipate that T118A mutation would perturb the conformational dynamics involving these two states. Furthermore, these two states are kinetically connected to other states and thus the mutation may also affect other states (Fig. 5). To investigate the variations of the conformational dynamics caused by the mutations, MSM need to be constructed based on the extensive MD simulations of GlnBP with the specific mutation, which is beyond the scope of current study. We are interested in exploring this in our future work. But it's worthy to note that the role of Thr118 in manipulating the ligand binding affinity is pinpointed by two new states (S1 and S1' states) showing small domain binding. The mutagenesis experimental validation can serve as a proof of the new conformational states discovered by the MSM.

Accordingly, we have added the following discussion in the "Discussion" section in page 16:

"*MSM constructed based on extensive MD simulations of GlnBP with the specific mutation would help to elucidated the detailed variation of protein dynamics caused by the mutation.*"

<u>Response to Reviewer #2's Comments:</u>

Here the authors combine MD simulations, Markov state model (MSM) analysis and experiments to obtain insight into the conformational dynamics of ligand-bound glutamine binding protein (GlnBP). Their integrated analysis indicates that ligand-bound GlnBP is highly flexible with multiple metastable binding sites. Interestingly, these present a more complex energy landscape than the one in the absence of ligand. The conformational ensemble of GlnBP presents different binding affinities and of note, complex transition kinetics, suggesting a concerted ligand binding mechanism. The authors then carry out single molecule FRET and mutagenesis experiments. These confirm their MSM-derived conformational ensemble and their binding mechanism.

I like this paper since it is not only comprehensive, meticulous and very carefully done; in particular, it elucidates the linkage between protein dynamics and ligand binding, clearly showing that protein dynamics is essential for ligand binding. They also propose a complex regulatory network that may underlie the apparent binding affinity.

This is fundamental research into the basic science underlying protein conformational behavior, its dynamics and function. The paper is very carefully prepared and is very clear. Readers should find it of interest.

Reply: We would like to thank this reviewer to acknowledge our work as "*a fundamental research into the basic science underlying protein conformational behavior, its dynamics and function*". He/She thinks our study has clearly shown that "*protein dynamics is essential for ligand binding*".

Response to Reviewer #3's Comments:

The authors present a computational study of the glutamine binding protein, and through a Markov State Model for the ligand-bound and unboud states propose a scheme for a ligand binding scheme that is coupled to internal protein dynamics. The study is complemented by FRET and mutagenesis experiments. Although in principle interesting, the study suffers from a major flaw based on which I cannot recommend it for publication.

The ligand binding affinity for each state is estimated based on a Poisson-Boltzmann surface area (MM-PBSA) method. Although the experimental affinity for the ligand is reported in the high nanomolar range (which corresponds to about 40 kJ/mol), the computationally reported affinities are up to > 300 kJ/mol (Fig. 3). This large discrepancy for the computational affinity precludes any mechanistic interpretation or comparison to experiment..

Reply: This reviewer found our study in principle interesting, but also expressed his/her concern about the discrepancy between the ligand affinity data obtained from wet experiments and computation using Molecular mechanics Poisson–Boltzmann surface area (MM-PBSA) method. We would like to thank the reviewer for bringing up this question and we agree with the reviewer that there is large discrepancy between experimental and computational binding affinities. To address his/her concern, we have discussed the reasons for the discrepancy (see below for details) and demonstrated that the calculated binding free energies in Fig. 3a are not comparable with the experimental binding affinity in Fig. 6g. We hope our explanation and modifications can help avoid confusion.

Specifically, we attribute the discrepancy to the following two aspects:

First, this discrepancy can be originated from that we did not consider entropy's contribution when calculating the absolute binding free energies ($\Delta G_{binding}$) using MM-PBSA method. Although entropy is one component in characterizing the absolute binding free energies, estimation of entropy is always time-consuming and displays a large magnitude of standard error. Therefore, the entropy term is simply ignored in many practical applications. Even so, MM-PBSA is usually considered as a reliable approach in estimating the relative binding free energies ($\Delta(\Delta G_{binding})$) (*Chem. Rev.* 119, 16, 9478-9508 (2019); *Front. Mol. Biosci.* 4(87) (2018); *Phys. Chem. Chem. Phys.* 16, 16719 (2014)). Actually, MM-PBSA has become one of the most popular methods in estimating relative binding free energies ($\Delta(\Delta G_{binding})$) and widely used in biomolecular studies as well as computational drug design (*Mol. Pharmaceutics* 17, 132−144 (2020); *ACS Catal.* 9, 2381−2394 (2019); *Chem. Eur. J.* 25, 10350–10358 (2019); *Expert Opin. Drug Dis.* 10(5):449-461(2015)). A previous study based on more than 1,800 protein–ligand crystal structures also demonstrates MM/PBSA is suitable for individual-target-level binding free energy ranking (*Phys. Chem. Chem. Phys.* 16(31):16719-16729 (2014).). Therefore, we think MM-PBSA serves as a proper method and fits well with our purpose to compare the relative protein-ligand binding strength among different macro-states, in order to correlate binding affinity with protein dynamics.

Second, this discrepancy also comes from that the reported binding free energy in Fig. 3a is for each macro-state, while experimental apparent binding affinity in Fig. 6g is an ensemble-averaged quantity. Therefore, the binding free energies of each macro-state (Fig. 3a), which could not be measured by experimental method, is not comparable with the apparent K_d from an ensemble of protein-ligand conformations (Fig. 6g).

In summary, although the $\Delta G_{binding}$ of each macro-state in our calculation in Fig. 3 is not comparable with the experimental apparent binding affinity in Fig. 6g, the relative binding free energies ($\Delta(\Delta G_{binding})$) can shed light on the relative binding strength between protein and ligand in each state. This would allow us to investigate the interplay between binding affinity and protein dynamics.

To avoid the confusion, we have changed the legend of y-axis from $E_{binding}$ to $\Delta G_{binding}$ in Fig. 3a of the revised manuscript:

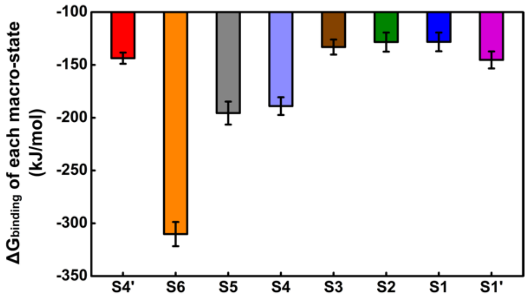

Fig. 3 (a) Ligand binding free energies of macro-states are estimated by MM-PBSA method

Furthermore, we have added two review papers (*Chem. Rev.* 119, 16, 9478-9508 (2019) and *Front. Mol. Biosci.* 4(87) (2018)) from the above citations as well as the following sentence in page 6 of the revised manuscript to clarify this point:

"*This method has been widely applied in areas of biomolecular studies and computational drug design to estimate the relative binding free energies. We note the binding free energy of individual macro-state could not be measured by experimental method, but it can shed light*

on the relative binding strength between protein and ligand in each state. Accordingly, we …"

REVIEWERS' COMMENTS:

Reviewer #1 (Remarks to the Author):

The authors have addressed all my concerns.

Reviewer #3 (Remarks to the Author):

Unfortunately my concern about the reported binding free energies has not been satisfactorily addressed. The authors replied twofold:

(1) "First, this discrepancy can be originated from that we did not consider entropy's contribution when calculating the absolute binding free energies s (ΔGbinding) using MM-PBSA method. Although entropy is one component in characterizing the absolute binding free energies, estimation of entropy is always time-consuming and displays a large magnitude of standard error. Therefore, the entropy term is simply ignored in many practical applications." That the computation is time-consuming and error-prone is a poor justification for neglecting the contribution. Only a calculation that would suggest that the contribution is negligible could serve as such a justification. But the enormous discrepancy between the experimental value of ~40 kJ/mol and the computational value of > 300 kJ/mol in fact suggests the contrary.

(2) "Second, this discrepancy also comes from that the reported binding free energy in Fig. 3a is for each macro-state, while experimental apparent binding affinity in Fig. 6g is an ensemble averaged quantity." Again, this is highly misleading, as the computational findings could be readily ensemble-averaged to yield a value comparable to experiment. The strongest binding macrostate S6 would contribute most to that average, therefore yielding an overall estimate in the range of 200-300 kJ/mol, in clear disagreement with the experimental value of ~40 kJ/mol.

The legend change from Ebinding to ΔGbinding in Fig 3, or the citation of other papers using the same methodology do not help to resolve this issue.

Response to Reviewer#1's Comments:

The authors have addressed all my concerns.

Reply: We thank the reviewer for his/her time and efforts in making very thoughtful comments on our manuscript. His/her comments have greatly helped us to improve our manuscript.

Response to Reviewer#3's Comments:

Unfortunately my concern about the reported binding free energies has not been satisfactorily addressed. The authors replied twofold:

(1) "First, this discrepancy can be originated from that we did not consider entropy's contribution when calculating the absolute binding free energies s (ΔGbinding) using MM-PBSA method. Although entropy is one component in characterizing the absolute binding free energies, estimation of entropy is always time-consuming and displays a large magnitude of standard error. Therefore, the entropy term is simply ignored in many practical applications." That the computation is time-consuming and error-prone is a poor justification for neglecting the contribution. Only a calculation that would suggest that the contribution is negligible could serve as such a justification. But the enormous discrepancy between the experimental value of ~40 kJ/mol and the computational value of > 300 kJ/mol in fact suggests the contrary.

(2) "Second, this discrepancy also comes from that the reported binding free energy in Fig. 3a is for each macro-state, while experimental apparent binding affinity in Fig. 6g is an ensemble averaged quantity." Again, this is highly misleading, as the computational findings could be readily ensemble-averaged to yield a value comparable to experiment. The strongest binding macrostate S6 would contribute most to that average, therefore yielding an overall estimate in the range of 200-300 kJ/mol, in clear disagreement with the experimental value of ~40 kJ/mol.

The legend change from Ebinding to ΔGbinding in Fig 3, or the citation of other papers using the same methodology do not help to resolve this issue.

Reply: We are sorry that the reviewer's concern about the calculated binding free energies has not been satisfactorily addressed in our previous response, and we would like to thank the reviewer for this comment. As pointed out by the reviewer in point (2), the experimental measurement of binding affinity is 202nM, which corresponds to -63.0 kJ/mol when T=291.15 K (the temperature for ITC measurement). Following the reviewer's suggestion, we have calculated the ensemble-averaged binding free energy as -197.1±4.1 kJ/mol, which indeed exhibits a large discrepancy from the experimental binding affinity obtained from ITC measurement. We attributed this discrepancy mainly to the conformational entropy, the contribution of which is not considered in the MM-PBSA calculations for the absolute binding free energies ($\Delta G_{binding}$) (*Chem. Rev.* 119, 9478 (2019); *J. Chem. Inf. Model.* 54, 1951 (2014)). Since the entropy's contribution of the different metastable states with the same ligand are usually similar (*Chem. Rev.* 119, 9478 (2019)), it is reasonable to leave out the entropy term in the calculations when the relative binding affinity rather than the absolute value is needed. In fact, MM-PBSA has been a popular method and widely used in estimating the relative binding free energies ($\Delta(\Delta G_{binding})$) in many protein-ligand or macromolecular systems due to its reasonable accuracy and efficiency (*Chem. Rev.* 119, 16, 9478-9508 (2019); *Front. Mol. Biosci.* 4(87) (2018); *Phys. Chem. Chem. Phys.* 16, 16719 (2014)). The

reliable ranking performance of MM-PBSA serves the purpose of our current work, which focuses on exploring the interplay between the protein dynamics and ligand binding. In this regard, the relative ligand binding strength in each metastable state, rather than their absolute values, estimated by MM-PBSA method enables us to correlate the relative binding affinity with protein dynamics.

In the revised manuscript, we have inserted the following sentences to include the ensemble-averaged calculated binding free energy and discuss about the discrepancy between the calculated binding affinity and the experimental value. We hope these modifications can relieve the reviewer's concern.

In the "Discussions" section,

"*It is noted there exists a large discrepancy between the ensemble-averaged absolute binding free energy ($\Delta G_{binding}$) calculated using MM-PBSA method (-197.1±4.1 kJ/mol) and that obtained from the ITC measurement (202nM, corresponding to -63.0 kJ/mol at T=291.15K). One major source of this discrepancy is the conformational entropy, which is not considered in the MM-PBSA calculations. Since the entropy's contribution in different metastable states with the same ligand are usually similar, it is reasonable to leave out the entropy term in the calculations when the relative binding affinity rather than the absolute value is needed. Actually, MM-PBSA is popular in estimating the relative binding free energies ($\Delta(\Delta G_{binding})$) in many applications with reasonable accuracy and efficiency, which fits our purpose to correlate the relative ligand binding affinity of each conformational state to the protein dynamics in the current study*."

In the "Method" section,

"*The ensemble-averaged binding free energy contributed by all metastable states was computed as the weighted sum of the binding free energy of each microstate. The weight is determined by the population of the respective microstate. Mean and standard deviation were estimated by performing 1000 iterations of such calculations*."
